# More than just investment: Causality analysis between foreign direct investment and economic growth

**DOI:** 10.1371/journal.pone.0276621

**Published:** 2022-11-03

**Authors:** Shenali Nupehewa, Sachintha Liyanage, Deshan Polkotuwa, Madhurshi Thiyagarajah, Ruwan Jayathilaka, Anuja Lokeshwara

**Affiliations:** 1 SLIIT Business School, Sri Lanka Institute of Information Technology, Malabe, Sri Lanka; 2 Department of Information Management, SLIIT Business School, Sri Lanka Institute of Information Technology, Malabe, Sri Lanka; Universiti Malaysia Sabah, MALAYSIA

## Abstract

This study examines the causal nexus between Foreign Direct Investment (FDI) and the economic growth of seven (7) regions encompassing 117 countries. A more recent panel dataset over the period 2010–2020 was analysed using the Granger causality approach and panel VAR/block exogeneity test to conduct predictive analysis among the panel series. Wavelet coherence techniques too were adapted in bringing novelty and further justifications to the research in exploring the interaction effects of the variables, which are yet to be popularised in the studied discipline. The empirical results indicate the presence of bi-directional causality between FDI and economic growth globally and in the Asian region. In contrast, the causality is uni-directional in the American region. A non-directional causality was discovered in European, Oceanian, Mediterranean, and African regions, and the findings were consistent with the outcome of the wavelet coherence technique results. The study further classifies the regions into three cross-market categories such as developed, emerging and frontier markets. The results imply no causality for most developed and emerging economies in the regional analysis. Findings also provide insights for governments and policymakers worldwide to formulate policies on directing FDI flows and propositions for a host country to become a more conducive destination for FDI and accelerate economic growth.

## Introduction

Foreign capital, specifically Foreign Direct Investment (FDI), is surging worldwide due to the interconnection of national economies through international trade and capital transfers, boosting overall economic growth. This has been further fuelled by the rise of globalisation and amicable diplomatic ties nations have developed, in addition to well-connected financial markets. However, despite the extensive past literature on the relationship between FDI and economic growth in both developed and developing countries, conflicting results were observed throughout [1].

According to Kalai and Zghidi [2] and Belloumi [3], the significant role of FDI in host countries is to facilitate the provision of funds for domestic investment. However, its potential extends critical macroeconomic concerns such as assessing the host country’s export capacity, allowing countries to boost their foreign exchange gains, and improving job creation, knowledge transfers, and economic growth [4]. These favourable influences have fostered the funds flow between nations. As per the Investment Trends Monitor of the United Nations Conference on Trade and Development (UNCTAD), the global FDI flows rebounded strongly in 2021, increasing by 77% from $929 billion in 2020 to an expected $1.65 trillion exceeding their pre-COVID-19 level.

The interaction between FDI and economic growth has recently garnered considerable attention from researchers and policymakers in the host countries. Iamsiraroj and Ulubaşoğlu [5] provide an overview of the empirical findings on the FDI-growth relationship; they reported that 43% of the 108 empirical studies reviewed had indicated a positive and significant effect while 17% indicated a significant negative effect and 40% of the studies reported a statistically insignificant effect. These statistical observations signify the necessity of FDI to aid a nation’s growth. Duarte, Kedong [6] and Banday, Murugan [7] show that many nations worldwide have altered their FDI policy criteria to attract FDI by providing incentives such as tax deductions.

Extant literature concludes the relationship to be uni-directional, bi-directional, or non-directional [8–10] in terms of causality between FDI and economic growth. Most past studies demonstrate that FDI and economic growth have a positive causal relationship in the short run, long run or both. In a similar study by Frimpong and Oteng Abayie [11] found no relationship between FDI and Ghanaian economic growth. Studies by Afolabi and Bakar [12] and Sunde [8] on Nigerian and South African economies revealed a bi-directional relationship, while studies on the Asian and European economies by Palamalai Srinivasan, M. Kalaivani [13] and Pradhan, Arvin [14], respectively, revealed a bi-directional linkage. In contrast, Onafowora and Owoye [15] discovered a uni-directional link in the American region, highlighting that a consensus was not reached concerning this relationship and the direction over a wide range of countries.

Whilst the hitherto literature emphasises mixed results for the causality between FDI and economic growth, it also justifies the need for more detailed research in the subject domain. Though several studies have investigated the relationship between FDI and economic growth, the direction of causality between these variables is particularly under-examined, though it cannot be underrated. Moreover, it is crucial to examine the relationship between FDI and economic growth to identify whether FDI fueled the swift economic growth of the considered regions, or whether a reciprocal relationship existed between economic growth and FDI,—which was not sufficiently addressed in the extant literature.

The study’s main objective is to verify the causal link between FDI and economic growth by capturing seven (07) regions and a more refined technical analysis.

Consequently, the present study substantially contributes to the existing literature on five fronts: firstly, it investigates the causal relationship between FDI and economic growth across six regions and multiple countries in a global context using a more recent panel dataset that covers the period from 2010 to 2020; secondly, it deploys a bivariate Granger causality model and panel VAR/block exogeneity test on the acquired data, which has been proven to be superior to conventional panel data analysis approaches; thirdly, conducts an in-depth exploration of the dynamic causal relationship between FDI and economic growth for regions globally in recent years. The novelty of this study is that, to the best of the authors’ knowledge, it is the pioneer study done by local researchers that uses current panel data methodology to observe the causal relationship of the study variables across world regions. Fourthly, this is the first paper to bring the wavelet coherence technique that offers more intuitive understanding of the linkages between the considered variables signifying short-term or long-term reactions. This is yet to be popularised in the discipline of study, to further justify the interaction effects of the variables together with the Granger causality test, which improves the originality of the study. This will assist in understanding the direction of the causal relationship between these macroeconomic variables and aid the governments, stakeholders, private institutions, academic scholars, and policymakers in duly integrating their FDI policies into macroeconomic objectives for all-inclusive, sustainable economic growth and development. Fifthly, this study is the initial panel study to extensively examine the dynamic causal linkage between FDI, net inflows, and economic growth for regional cross-markets besides the comparative regional analysis and enables to understand the influence of cross-market differences on FDI and GDP within the scope of developed, emerging and frontier markets.

The subsequent sections of this paper outline the empirical review of the literature followed by model specification, estimation approaches, analysis and discussion of the findings, and finally reaching the conclusions and policy implications.

## Literature review

**Fig 1:**
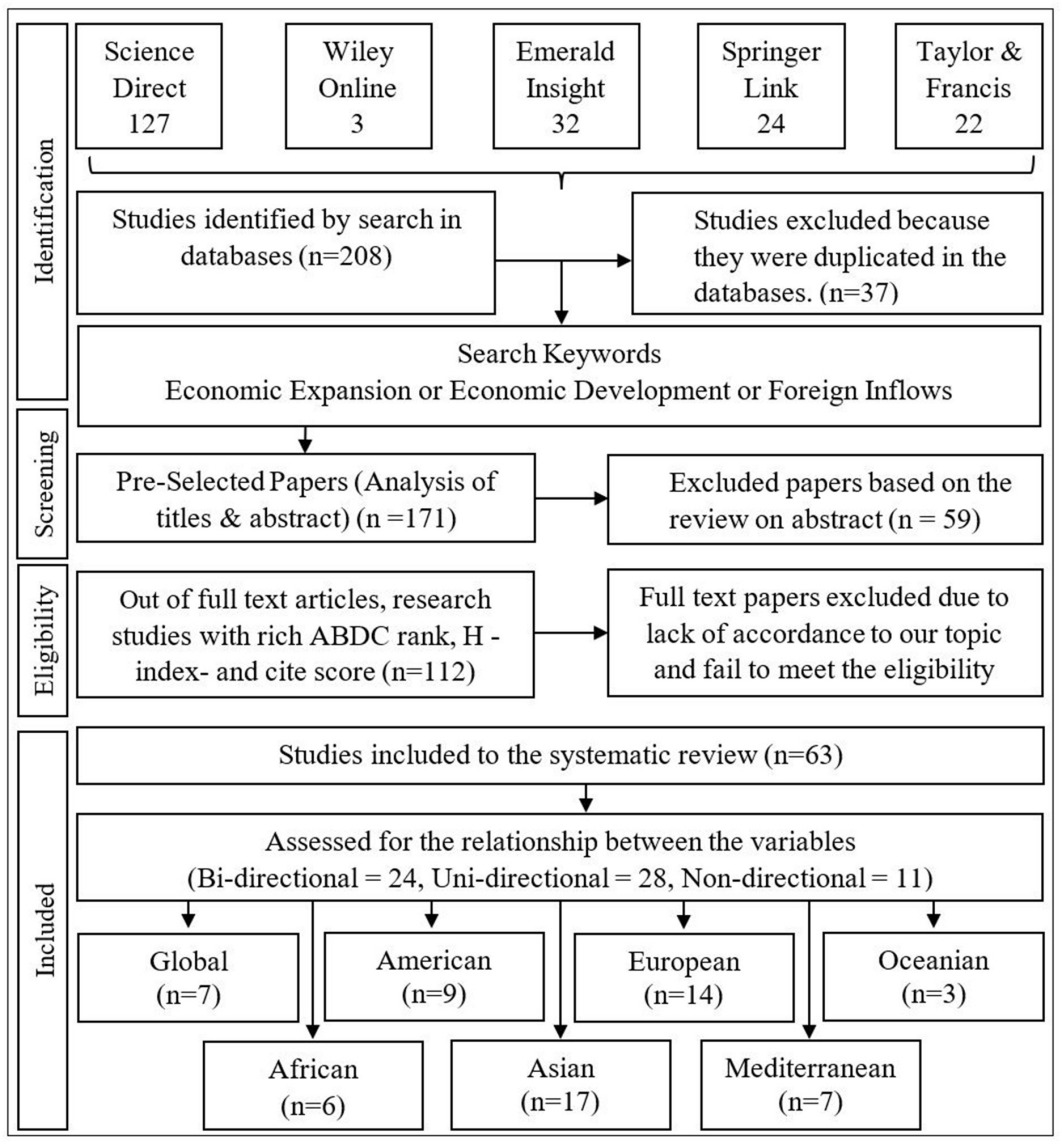
Literature search flow diagram. Source: Authors’ Illustration.

This study centred the literature review on 63 initially identified articles through a comprehensive and detailed literature search shown in Fig 1. Several reputable research databases including Science Direct, Emerald Insight, Taylor & Francis Online, Wiley Online Library and Springer Link, were referred to among others.

The search strategy was divided into four parts, namely: (i) identification, (ii) screening, (iii) eligibility, and (iv) inclusion. In the identification step, several relevant papers were explored using keywords. Initially, out of 208 research papers shortlisted, 37 were excluded due to duplications. The researchers directed the remaining 171 papers to the screening stage and read and reviewed the abstracts of the research papers. The studies deemed to have insignificantly contributed or were unrelated to the selected topic were excluded resulting in 59 exclusions.

The remaining 112 research papers were further screened in the eligibility step. The ABDC rank, Scopus, CiteScore, and Scimago H-index of journals pertaining to published papers were checked. The papers that have slight relevance to the topic and papers published in low-ranking journals were excluded resulting in 49 exclusions. Subsequently, the remaining articles were classified into three (03) main categories considering the relationship of main variables as bi-directional causality, uni-directional causality and non-directional causality.

The selected 63 papers were further categorised into seven topics, namely global (research conducted on many countries without being based on a specific geographical categorisation) and identified regions: Asian, African, American, European, Oceanian, and the Mediterranean. S1 Appendix comprises studies concerning the causal relationship between FDI and economic growth, in terms of the research country, period under consideration, the methodology employed and the conclusions reached.

The literature pertaining to the relationship between FDI, and economic growth was discussed to establish a solid scientific background for the study. This section will present an extensive review of the literature to emphasise research findings related to this topic, identify their limitations and knowledge gaps and highlight the contribution of the research.

According to the study by Mahembe and N [9], FDI contributes directly and indirectly to economic growth, and the host country’s growth may attract more FDI through exogenous and endogenous growth theories. Here, FDI has been found to affect economic growth in two ways: firstly, through technological spillovers, by encouraging the adoption of new technologies in the manufacturing process; and secondly, by stimulating the knowledge transfer, both through labour training and skill acquisition, as well as introducing alternative management practices. Studies done by Barro and Sala-I-Martin [16], De Jager [17], Fan [18] and Romer [19] have also shown consensus towards these findings.

The causal relationship between FDI and Gross Domestic Product (GDP) growth could move either way. According to McKinnon [20], the major obstacle to economic growth in emerging countries is the paucity of technology. Furthermore, researchers have found that FDI probably boosts the host country’s economic growth through knowledge and technology transfers and spillover effects [6]. From a strategic stance, FDI is more likely to provide nontangible assets, such as advanced technology as well as beneficial spillover effects to domestic enterprises. Consequently, foreign investors may boost productivity in the receiving economy, and FDI can be considered a growth driver.

The causality could also move in the opposite direction; rapid GDP growth often results in a massive capital resource gap in a host nation. Since FDI is one of the main sources of capital [21], the host governments attempt to alter policy frameworks to attract sufficient foreign investors. More importantly, the host country’s rapid economic growth will boost foreign investors’ confidence and improve economic growth, thereby improving foreign investors’ investment environment. Rapid economic expansion, accompanied by rising per capita income, will pave the way for enormous profit prospects in the host country. The output growth, indeed, is considered one key determinant for FDI inflows to a host country and this debate is often called “the market-size hypothesis” or “the growth-driven FDI hypothesis” [22, 23]. This hypothesis emphasises the importance of growing market size, and the penetration of foreign markets is a major motive for FDI [23]. Hence, output growth is a vital element in reflecting the market size in a host country, which acts as a positive influence to attract more FDI to a nation.

The latent literature consistently highlights three main conclusions on the relationship between FDI and economic growth; uni-directional, bi-directional, or non-directional. A discussion on the existing literature pertinent to the relationships of the main variables studied in this research is discussed region-wise henceforth.

### Global

FDI flows grew dramatically globally in the last two decades. This is because many countries view FDI as a critical component of their respective economic growth strategies. Several studies consider the possible relationship between FDI and economic growth globally. Similarly, a study for 19 Eurozone countries by Pradhan, Arvin [14] and a study by Mahmoodi and Mahmoodi [24] comprising eight Eurozone and eight Asian developing nations also revealed bi-directional causality between FDI and economic growth. A recent study by Qureshi et al. [25] observed a bi-directional causality in 54 developed and developing countries. All continents were considered in this study, although a developing country from Oceania was not included. Ahmad, Draz [26], Banday, Murugan [7] and Saidi, Mani [27] too proved the above causality for some countries, including the Association of Southeast Asian Nations (ASEAN) and the BRICS nations: Brazil, Russia, India, China and South Africa.

Iamsiraroj and Ulubaşoğlu [5] performed a meta-regression analysis and concluded that contemporaneous FDI strongly affects economic growth, and FDI is positively associated with countries with lower inflation and larger government size. The authors highlighted that the link is higher in single country investigations than in cross country examinations. Apergis, Lyroudi [28] discovered that FDI has a significant causal relationship with economic growth using 27 transitional economies of Europe and Central Asia. Jyun-Yi and Chih-Chiang [29] also found that FDI significantly and positively affects economic growth by analysing a dataset with 62 countries spanning every continent. Duttaray, Dutt [30] and Abbes, Mostéfa [31], proved the FDI-led growth hypothesis for multiple countries. Opposingly, Ahmad, Draz [26], Aslan, Altinoz [32] and Gupta and Singh [33] found uni-directional causality from economic growth to FDI. However, none of the selected studies has covered sectoral FDI contribution in a global context. Therefore, it is worthwhile to investigate the growth impact of various FDI types in their respective sectors, covering the global economy, by conducting a panel study.

### African region

A few studies conducted analysed FDI and economic growth in Africa. These revealed that FDI can be transformative for the region and is crucial for helping Africa towards long-term sustainability and economic growth.

Using the Autoregressive Distributed Lag (ARDL) bounds testing approach and the Granger causality test, Owusu [34] examined the relationship between FDI and economic growth in Namibia and found a two-way causal connection exists between the two variables in both short and long terms. A study on 25 African nations by Akadiri, Gungor [35] also confirmed the same conclusion using panel bootstrapping co-integration and the Granger causality.

A few studies have concluded that the said causality is uni-directional in African countries. Mahembe and Odhiambo [10] found that middle-income countries (MICs) of Southern African Development Community (SADC) states have a one-way causality from GDP to FDI. Although, when considering the low-income countries (LICs) of SADC, no evidence was found for causality in either direction. Sunde [8] deployed the ARDL bounds testing approach, Vector Error Correction Model (VECM), and the Granger causality approach on data from South Africa and found a uni-directional causality running from FDI to economic growth. In a more recent Kenyan study using the ARDL bounds testing approach, Odhiambo [36] found a uni-directional causality running from economic growth to FDI.

Moreover, non-directionality between FDI and economic growth was observed in Africa as evidenced by Tekin [37] in his study that addressed a sample of the least developed African countries. A similar non-directionality was evidenced in Tunisia by Belloumi [3], which contradicts the widespread belief that FDI can generate positive spillovers for a host country. Hence, it can be recommended to investigate critically the barriers of FDI in fuelling economic growth in the African region.

### American region

The causal relationship between FDI and economic growth requires much attention in the American region. Contemporary studies done by Al Nasser [38] and Owusu-Nantwi and Erickson [39] concluded that in South American countries, economic growth could attract FDI, which in return would accelerate economic growth. This highlights the presence of a bi-directional causality between FDI and economic growth in South American countries. Similar corroborating evidence was found in a study by Onafowora and Owoye [15], which revealed a bi-directional Granger causality between FDI and economic growth in the Bahamas.

Contradicting the findings of these studies, Al Nasser [38] found a uni-directional causality exists between the two variables, running from economic growth to FDI. Dinç and Gökmen [40] discovered that in Brazil, causality runs from a change in FDI to change in economic growth, in terms of GDP. Using the Granger causality test, Onafowora and Owoye [15] found that in Barbados and the Dominican Republic, a uni-directional causality exists from output growth to FDI, and from FDI to economic growth in the Caribbean nations of Jamaica and Trinidad and Tobago.

Furthermore, Naguib [41] and Chen-Chang Lo, Chi [42] discovered that in Haiti, FDI and economic growth do not significantly influence each other. In addition, the study by Dinç and Gökmen [40] found no positive causality between the two variables in the short run. In addition, there is a noticeable deficiency in research relevant to the topic on the majority of Latin American countries and North American non-sovereign states. Since the American region consists of countries with varied income levels (i.e. highly developed, middle-income, and LICs), the interactions between FDI and economic growth should be investigated with particular attention so as to allow customised policy recommendations and employ more robust econometric models.

### Asian region

A vast majority of empirical studies have focused on the effects exerted on economic growth along the causal link between FDI and economic growth. Palamalai Srinivasan, M. Kalaivani [13] found that in South Asian Association for Regional Cooperation (SAARC) countries, a bi-directional causality is evident between FDI and economic growth except in India, where a long run one-way causality running from GDP to FDI. However, this study recommends that further research should be conducted using quarterly data to further observe differences in causalities. Studies done by Anwar and Nguyen [43], Balamurali and Bogahawatte [44], Kaur, Yadav [45] and Liu, Burridge [46] analysed the causal relationship between FDI and economic growth in Asia and also concluded causality to be bi-directional.

Two studies by Iqbal, Shaikh [47] and Chandio, Mirani [48] investigated the causality that overall FDI and agricultural sector FDI have with economic growth, respectively, in Pakistan. The findings revealed a bi-directional causality between FDI and economic growth in both the short and long run. Liu, Shu [49] employed a VECM on a sample of 09 Asian countries and reached the same conclusion. These results also indicated two-way causality between all variables, including inward FDI and economic growth. Studies by Zhao and Du [50] and Dash and Sharma [51] indicated similar results, although the result of the former study was not highly significant. Chowdhury and Mavrotas [52] examined the causal relationship between FDI and economic growth in Chile, Malaysia, and Thailand using the Toda-Yamamoto test for causality. This study concluded a bi-directional causality for two countries except for Chile, where a uni-directional causality running from GDP to FDI was reported.

Contrary to these studies, some research papers have concluded that FDI and economic growth have a uni-directional causality in the Asian region. Hsiao and Hsiao [53] found a Granger causality running from FDI to GDP and similar results were also observed in the following studies that employed different analysis techniques: Jayachandran and Seilan [54] and Goh, Sam [55] used cointegration analysis and Feridun and Sissoko [56] used Granger causality and Vector Auto-Regressive (VAR) models [55, 56]. However, a recent study by Sarker and Khan [57] revealed a uni-directional causality running from GDP to FDI in Bangladesh, which contradicts the findings of the previous ventures.

In addition, a Singaporean study by Akalpler and Adil [58] and a Cambodian study by Sothan and Zhang [59] discovered no causal relationship between FDI and economic growth. Both studies have incorporated VECM and the Granger causality test, covering the same period (1980–2014). Further research is worth examining the key determinants enhancing the relationship between FDI and economic growth.

### European region

The empirical evidence of the relationship between FDI and economic growth is inconclusive in the European region. Surprisingly, no study has found bi-directional causality between FDI and economic growth in Europe. However, Moudatsou [60] found that in the European Union (EU), FDI inflows from 1980 to 1996 have significantly affected economic growth. In addition, Dritsaki, Dritsaki [61], Cicak and Soric [62] have proven a uni-directional causality running from FDI to economic growth in some European countries. Studies by Varamini and Kalash [63] for 10 emerging European economies, Kuo, Lai [64] for Germany and Hobbs, Paparas [65] for Albania proved a reverse uni-directional causality from economic growth to FDI.

In contrast to these studies, Nath [66] concluded that there is no significant effect on economic growth by FDI in general. However, according to this study, FDI becomes a significant determinant of economic growth when the effects of trade and domestic investment are controlled. In addition, Golitsis, Avdiu [67] found no causal relationship between FDI and economic growth and other study variables. These noneconomic externalities of FDI need to be considered in future research, specifically in the European region.

### Mediterranean region

Determination of the causal patterns between FDI and economic growth has important implications for a developing region and the causality between FDI and economic growth in the Mediterranean region has been substantiated by numerous empirical studies.

A study in Turkey done by Gunaydin and Tatoglu [68] and an Iranian study by Yazdi, Soheila [69] found strong evidence for bi-directional Granger causality for FDI and economic growth. In addition, several hypotheses were tested in these studies concerning the causal relationship between FDI and economic growth. Opposing the previous conclusions, Kalai and Zghidi [2] and Alshehry [70] discovered a long-run uni-directional causality from FDI to economic growth in some countries of the region. Ibrahiem [71] further revealed long-run uni-directional causality from FDI to economic growth in Egypt using ARDL bounds testing approach and the Granger causality test.

Asheghian [72] concluded that a causal relationship does not exist between FDI and economic growth in Iran’s GDP per capita. Therefore, further studies can be conducted to find the causal relationship of FDI and economic growth by analysing all countries in the region which currently hold conflicting and divergent findings.

### Oceanian region

In the FDI-growth literature, empirical studies have so far yielded mixed results in the Oceanian region. Jayaraman and Choong [73] concluded that FDI and economic growth show a bi-directional causal link in Fiji, using Granger non-causality test and VECM.

Besides, Iyer, Rambaldi [74] proved that in Australia, all three foreign investment categories, namely: FDI, foreign portfolio investment, and other foreign investments, have a uni-directional causal relationship with GDP in the short run, while only FDI has long-run effects on GDP. Pandya and Sisombat [75], however, indicated evidence for the absence of a causal relationship between FDI and economic growth in Australia. This study also recommended conducting a micro analysis on the different sectors that attract FDI in Australia and further investigating the complementary role of FDI in expanding the economy of the Oceanian region.

In addition, some country-specific studies have identified potential determinants of FDI such as trade openness [76–80] market sizes [77–81], labour costs, human capital [77, 80], infrastructure availability [77, 79, 80], industry value added, government consumption, telephone mainlines per 100 of population, financial development, service value-added, inflation and commercial energy use per capita. Moreover, expected years of schooling, political stability [76], exchange rates, tax rates, tax depreciation, tax holidays, trade barriers, gross domestic investment, gross capital formation, technology gap, economic freedom, research and development, and corruption [77] are considered. Further, these factors are considered key determinants: economic risk rating, financial risk rating, political risk rating, commodity price index, world stock market index, gross fixed capital formation [78], GDP growth, macro-economic stability, school enrollment rate [79], growth prospects and positive country conditions, government finance, rate of return on investment, policy measures [79], economic potential, labour market characteristics, technological progress, labour regulation, competitiveness and eligibility for Cohesion Fund [81].

However, these determinants have not been considered in this study due to the lack of data availability for some of the countries in the sample (i.e., Afghanistan, Vietnam, Dem. People’s Rep. of Korea, Maldives, Tajikistan etc.). Due to the higher sample size in the current study, it is unrealistic to encompass the stated determinants without obtaining blank values for several smaller economies.

## Materials and methods

### Data

This study created a macro panel model for 117 countries; specifically, 28 African, 18 American, 34 Asian, 27 European, 5 Mediterranean, and 5 Oceanian countries were analysed. The research data are confined to the period in which annual data are collected for the period 2010 to 2020 from the World Development Indicators published by the World Bank. The data file used for the study is presented in S2 Appendix.

The data utilised for the cross-market analysis covers 23 developed markets, 21 emerging markets and 16 frontier markets under separate regions, based on the Morgan Stanley Capital International market classification framework. The original sample size was reduced by considering the data availability. The list of countries used for the cross-market analysis is provided in S3 Appendix.

The paneled variable, economic growth in terms of GDP per capita is obtained by dividing the mid-year population by the gross domestic output. GDP is calculated as the total gross value added by all resident producers in the economy, plus any product taxes, minus any subsidies not included in the product value. It is estimated without considering the depreciation of manufactured assets or natural resource depletion and degradation. All collected data are in current US$ [82].

The other paneled variable, FDI net inflows, were taken as a percentage of GDP, where the FDI is defined as net inflows of funds used to acquire a long-term managerial stake (10% or more of voting stock) in a company that operates in a country other than the investors. The balance of payments represents the sum of equity capital, earnings reinvestment, other long-term and short-term capital. Data were extracted from [83], segmented by GDP, and displayed net inflows (new investment inflows less disinvestment) from foreign investors in the reporting economy.

### Methodology

Seemingly, the connection between foreign direct investment and economic development was carefully scrutinised by many countries. Notably, these studies have taken into the consideration many different econometrics methodologies such as regression, ARDL approach [71], and VECM [84].

However, the advantage of using the first-generation unit root tests assume that cross-section units are cross-sectionally independent [85].

The wavelet coherence approach assists to understand better the bi-dimensional causality of time and frequencies between FDI and economic growth. This approach simply unveils the concealed relationship in cyclical trends and patterns [86, 87].

Dumitrescu and Hurlin Granger causality approach has been optimal in determining the direction of the causal link. It is particularly useful for estimating a model with cross-sectional dependence and heterogeneity. Granger’s causality test performs significantly better when applied to panel data because more observations lead to more degrees of freedom and less collinearity between explanatory variables. Thus, it can be underlined that this analysis can give more handful information with respect to other causality analyses [88, 89].

However, this study employs a combination of methods like panel unit root, wavelet coherence and Granger causality techniques unlike previous research that only used a single method, i.e. panel data analysis.

To attain the purpose of the study which was to investigate the direction of causality between FDI and economic growth, the empirical methodology used is presented in multiple stages. Initially the unit root tests were performed to identify the stationary variables, followed by the wavelet coherence technique to observe time frequency dependence of the variables, in subsequence, a panel Granger causality to detect the dynamic relationship between the selected variables: economic growth in terms of GDP per capita and FDI net inflows.

### Panel unit root

The stationarity properties of the variables were determined using the panel unit root tests, which also helped avoid spurious analysis and check if data are not integrated at first order, that is, I(1). According to recent studies, panel-based unit root tests are more powerful than time series-based unit root tests [35]. In order to investigate the stationarity of the regressors, the study uses the unit root tests proposed by Levina, Lin [90] and Hadri [91]. Data was further differentiated to eliminate possible unit-roots.

### Wavelet approach

This study further utilised the wavelet approach in solidifying the findings of causality analysis and to observe the time–frequency dependence information between variables, which cannot be profoundly observed through the causality analysis. Dependency of time frequency between FDI, economic growth and other variables were obtained through wavelet approach which was premised on the work of Goupillaud, Grossmann [92]. The use of the technique in finance, economics and tourism related data was also incited by Pal and Mitra [93] and Wijesekara, Tittagalla [94], highlighting on the structural break which is often common in the discipline. Alola and Kirikkaleli [95], Adebayo [96], Kalmaz and Kirikkaleli [97], Adebayo [98], have further contributed in the development of the model, including the wavelet transformation of the two time series and the wavelet coherence at difference phases.

### Panel Granger causality test

Individual Wald statistics proposed by Dumitrescu and Hurlin [99] are used to estimate non-causality for heterogeneous panel data from *x*_t_ to *y*_t_. The econometric framework considers two covariance stationary variables, *x* and *y*, observed for T periods and N individuals. The analytical results of Dumitrescu and Hurlin [99] are based on Eq (1):

$$y_{i,t}=\alpha_{i}+\sum_{k=1}^{p}\gamma_{i}^{\left(k\right)}y_{i,t-k}+\sum_{k=0}^{p}\beta_{i}^{\left(k\right)}x_{i,t-k}+\varepsilon_{i,t}$$ (1)

where *α*_*i*_ represents the individual fixed effect across *i*, and $\gamma_{i}^{\left(k\right)}$ and $\beta_{i}^{\left(k\right)}$ are various coefficients of *y*_*i*,*t*−*k*_ and *x*_*i*,*t*−*k*_ for the individual *i*, respectively. The panel is balanced and lag orders *p* are identical for all cross-section units of the panel. *x*_*i*,*t*_ is said to Granger cause *y*_*i*,*t*_ if past values of *x*_*i*,*t*_ have a significant impact on *y*_*i*,*t*_ in addition to the past of *y*_*i*,*t*_.

To test for causality from FDI on economic growth, the Eq (2) can be specified as follows:

$$\Delta RGDPC_{i,t}=\alpha_{i}+\sum_{k=1}^{p}\gamma_{i}^{\left(k\right)}\Delta RGDPC_{i,t-k}+\sum_{k=0}^{p}\beta_{i}^{\left(k\right)}\Delta FDI_{i,t-k}+\varepsilon_{i,t}$$ (2)

Similarly, Eq (3) is applied to see whether there is a causal relationship between economic growth and FDI:

$$\Delta FDI_{i,t}=\alpha_{i}+\sum_{k=1}^{p}\gamma_{i}^{\left(k\right)}\Delta FDI_{i,t-k}+\sum_{k=0}^{p}\beta_{i}^{\left(k\right)}\Delta RGDPC_{i,t-k}+\varepsilon_{i,t}$$ (3)

where Δ*RGDPC* is real GDP per capita, Δ*FDI* is the FDI net inflows, *i* refers to the country (*i* = 1, ….,. *N*), *t* refers to the period (*i*t = 1, …, *T*), *p* refers to the lag, and *ε*_*i*,*t*_ refers to white-noise error terms. This method is vigorous when compared to other causality techniques [7]. In accordance with one of the key assumptions of this technique, all variables should be stationary at the level or after the first difference.

### Panel VAR/block exogeneity test

Most empirical studies in econometrics aim at analysing the relationship between variables by identifying whether a change in one variable can be predicted by a change in the previous values of another variable. Block Exogeneity Wald test [100] is performed to detect a chronological ordering of movements of variables. Hence, this approach is employed to investigate the variations in these two variables in each region separately [101].

## Empirical results and discussion

### Preliminary analysis

Overview of descriptive statistics of the key variables used in the study are presented in Table 1. In total, 1,287 observations have been recorded across all regions and on average, the mean GDP per capita for the regions analysed in this study are as follows: Globally–US$ 17,489, European region–US$ 38,493, Oceanian region–US$ 21,839, Mediterranean region–US$ 18,580, American region–US$ 14,674, Asian region–US$ 14,674, and African region–US$ 2,557, which is the lowest. Table 1 further depicts the mean value of net FDI inflows as a percentage of GDP for Asian, European, African, Oceanian, American and the Mediterranean regions, and can be signified as 7.98%, 5.03%, 5.03%, 4.21%, 3.83%, 2.81% and globally 5.58% respectively. Further, GDP per capita has a range from US$ 234.2 to US$ 118,823.6 with values of mean and standard deviation at US$ 17,488.5 and US$ 22,298.02, respectively. Between 2010 and 2020, FDI, net inflows to the selected global sample ranged between -40.08% and 280.13%, with an average of 5.58% and a standard deviation of 16.43%. The highest mean net FDI inflows level is recorded by the Asian region due to strong investment in services and manufacturing and high-tech sectors [102]. The lowest is recorded by the Mediterranean region. This might be due to aftermath of economic, social and political reforms caused by “Arab Spring” during the period [103]. Regarding the mean GDP per capita, the European region has the highest value in countries with higher working populations and labour productivity [104]. The African region has the lowest GDP value due to factors like government corruption, poor economic policies, and lack of openness to international markets [105].

**Table 1:** Descriptive statistics.

	Global	African	American	Asian	European	Mediterranean	Oceanian
**FDI inflows (% of GDP)**							
Obs.	1287	308	198	374	297	55	55
Mean	5.58	5.03	3.83	7.98	5.03	2.81	4.21
Std. Dev.	16.43	11.21	3.06	25.33	14.58	2.51	3.38
Min.	-40.08	-6.37	-3.99	-37.17	-40.08	-2.76	-0.04
Max.	280.13	103.34	16.04	280.13	108.42	11.13	19.59
**GDP per capita (US$)**							
Obs.	1287	308	198	374	297	55	55
Mean	17,488.5	2,557.64	14,674.2	13,793.6	38,493.3	18,579.9	21,838.86
Std. Dev.	22,298.02	2,926.9	15,089.7	18,107.9	27,464.2	12,767.9	23,593.1
Min.	234.2	234.2	1,172.1	493.7	2,124.7	2,422.5	1,604.2
Max.	118,823.6	16,390.8	65,279.5	85,075.9	118,823.6	43,839.3	68,150.1

Notes: Variables are in their level form; Obs., Std.Dev., Min. and Max. represent Number of observations, Standard Deviation, Minimum and Maximum, respectively.

Source: Authors’ Compilation

The Asian region has the highest standard deviation in net FDI inflows. This is due to the high dispersion of FDI inflows in the region. Asia contains countries with the highest FDI inflow levels such as Hong Kong, and Singapore as well as countries with lower FDI inflows, namely Iraq and Qatar. The lowest standard deviation is in the Mediterranean region. All the countries considered in this region have low levels of FDI inflows, hence, the dispersion and standard deviation are low. Likewise, Europe and Africa have the highest and lowest standard deviations in terms of GDP per capita.

Considering GDP per capita from 2010 to 2020, the European continent has recorded the highest value of US$ 118,824 whereas the African continent has the lowest GDP per capita of US$ 234. The maximum percentage of net FDI inflows is captured by the Asian region which is 280%, whilst the European continent has the lowest negative FDI inflows, reflecting disinvestments value of 40%, respectively. The findings also reveal that the minimum net FDI inflows in all regions have negative figures.

**Fig 2:**
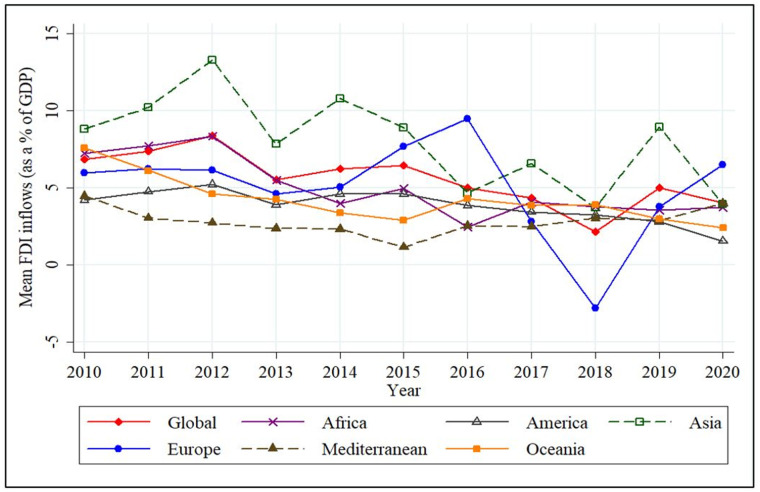
Region-wise mean FDI inflows, 2010–2020. Source: Authors’ Compilation based on World Bank [83].

The trends in the share of FDI in GDP for the countries are shown in Fig 2 from 2010–2020. The highest mean FDI inflows is recorded in the Asian region in 2012. A significant drop can be observed in the Asian FDI in 2016, but the scenario stabilised thereafter. Fig 2 shows a peaked curve in 2019 as East and South-East Asia took the lion’s share of foreign investment, accounting for one-third of worldwide FDI with inflows up to 2% and 11%, respectively. Following the global financial crisis, the FDI trends have shown slower growth, decreasing since 2013. The reasons behind this pattern, such as lower returns on foreign investment and disruptions in global value chains that are unlikely to change in the foreseeable future, can be disastrous. The European region had a rapid boost in 2016 because of the substantial investment from the United States of America (USA). A drastic decline in mean FDI inflows can be observed in 2017 and 2018. According to UNCTAD [106], this was due to the decision of multinational corporations in the USA to repatriate accumulated earnings after the USA imposed corporate income tax reforms following the global financial crisis and the global oil crisis. Brexit negotiations of 2017 may also have substantially affected FDI inflows into Europe because the United Kingdom’s (UK’s) claim to leave the EU is deemed that Europe is not profitable for foreign investors due to a persistent political crisis.

**Fig 3:**
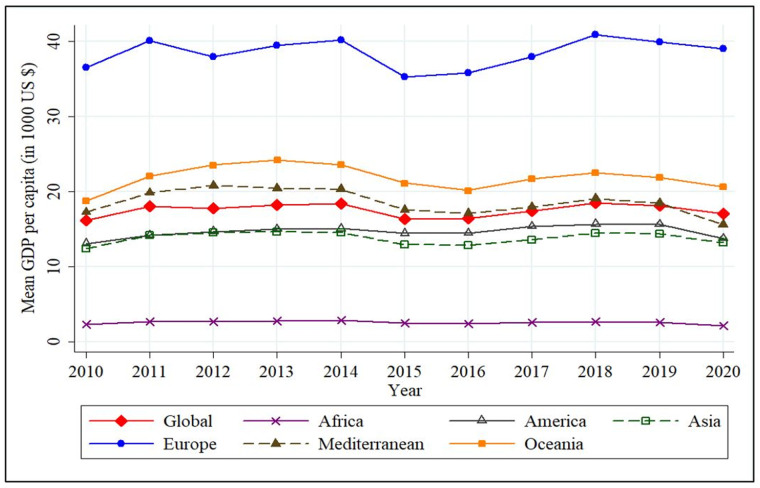
Region-wise GDP per capita, 2010–2020. Source: Authors’ Compilation based on World Bank [82].

Fig 3 indicates the graph of variations in the global mean GDP per capita and for the six individual regions over the 2010–2020 period. The highest mean GDP per capita can be observed in the European region. Two European countries, Luxembourg and Ireland, recorded the highest and the second highest GDP per capita in the decade, respectively. Luxembourg’s high GDP per capita can be explained by an abundance of foreign employees who do not reside in the country. It was also identified that Ireland’s high GDP per capita is partly due to the ownership of assets of large multinational organisations [107]. The lowest mean GDP per capita is recorded in the African region and the main factors contributing to this include political instability, diseases, and despotism [108, 109]. The African nation of Burundi has the lowest GDP per capita recorded in the time period considered. Moreover, a decline in mean GDP per capita can be observed for all regions as well as globally in 2015. This resulted from “demographic cliff” ICIS [110], where the number of low-spending, low-earning people over the age of 55 years worldwide surpassed 1 billion.

### Unit root results

The results of the stationarity tests are reported in Table 2. According to the Levin-Lin-Chu test, the null hypothesis is that the panels contain the unit root, while the alternative hypothesis is stationary. The adjusted t-statistic is employed at all the testing levels. The estimated p-values of the variables are compared to alpha (α) values of 1%, 5%, and 10%, respectively. The results reveal that both the variables appear to have some unit-roots at all testing levels but become stationary at first differences.

**Table 2:** Unit root test results.

Variables	FDI net inflows		GDP per capita	
	Level	First Difference	Level	First Difference
**Levin Lin Chu**				
Global	-10.2681***	-20.0267***	-6.4465***	-12.7934***
Africa	-4.6343***	-5.8107***	-3.3995***	-7.9290***
America	-0.1424	-9.0756***	-2.3549***	-1.0774
Asia	-4.4517***	-8.6768***	-3.1646***	-6.5811***
Europe	-5.1655***	-7.0146***	-4.4961***	-8.0948***
Mediterranean	-0.3021	-1.9012**	-0.8580	-2.4150***
Oceanian	-11.3204***	-13.9647***	-2.3293***	-2.5642***
**Hadri LM**				
Global	17.4277***	-6.0497	36.1851***	-0.5012
Africa	4.7803***	-2.7900	11.3963***	4.6853***
America	0.2459	-1.5217	15.8520***	1.6365*
Asia	12.0680***	-4.1438	20.3904***	1.2463
Europe	8.6898***	-0.9846	17.8813***	-1.6952
Mediterranean	7.1297***	0.9404	3.7792***	1.0541
Oceanian	1.5317*	0.8893	3.3396***	1.1367

Note:

*Reject H_0_ at a 10% level of significance;

**Reject H_0_ at a 5% level of significance;

***Reject H_0_ at a 1% level of significance

Source: Authors’ calculation.

The Hadri Lagrange Multiplier (LM) test confirms the results from the previous test, with the null hypothesis indicating that the panels are stationary and the alternate hypothesis indicating that some panels contain unit-roots. The results of the Hadri LM test indicate that the p-values of GDP and FDI at the first difference are greater than the significance levels of 1%, 5% and 10%, respectively. Therefore, failing to reject the null hypothesis implies that the panel data is stationary. Thus, it can be derived that both the micro panel variables are integrated at the first order, i.e., I(1) and are stationary at all testing levels.

### Wavelet coherence

Prior to analysing the findings of interaction effects through Granger causality, the wavelet coherence was deployed to instantaneously detect the correlation and causality between the FDI and other regressors, including economic growth. Interpretation of wavelet coherence requires analysing the area of influence depicted in the grey cone plotted with of R software. The level of significance is depicted by the thick black line determined via the Monte Carlo Simulations and Table 3 encapsulate interpretation guide as per Pal and Mitra [93], Adebayo [96], Kalmaz and Kirikkaleli [97], and Adebayo and Beton Kalmaz [111].

**Table 3:** Wavelet coherence interpretation.

Direction of arrows	Interpretation
⭧	FDI leads (causes) the GDP: (In phase)
⭨	GDP leads (causes) the FDI: (In phase)
⭦	FDI leads (causes) the GDP: (Anti phase)
⭩	GDP leads (causes) the FDI: (Anti phase)
Short term	0–8
Medium term	8–32
Long Term	32–64
Low frequency	0.0–0.3
Medium frequency	0.3–0.7
High frequency	0.7–1.0

Sources: Authors’ compilation.

The direction of the arrows will indicate whether the variables move in phase (rightward arrow indicating a positive correlation), or anti-phase (leftward arrow indicating a negative correlation) and the cold (blue) regions of the figure indicate no correlation.In contrast, the warm (red) regions depict the analysed variables are correlated.

**Fig 4:**
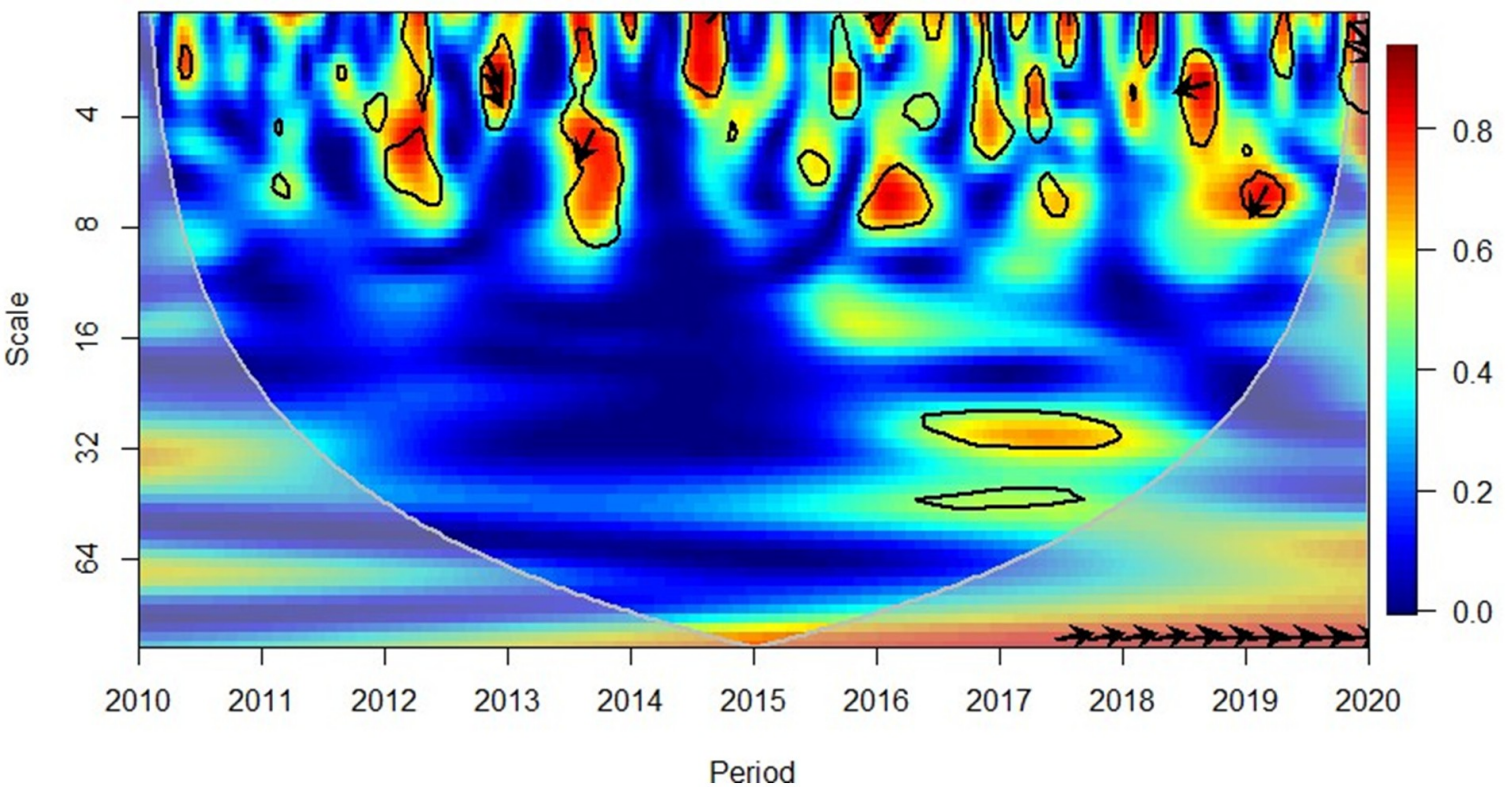
Africa region, GDP and FDI. Source: Authors’ compilation using R-Software.

In 2013 (Fig 4), in the short-term (high frequency) depicted a positive correlation between GDP and FDI in the African region, where the rightward down arrows indicate that the GDP led FDI. It was worthwhile to note that in the subsequent year (in 2014), a change in pattern is reflected in the short-term, (high frequency) with leftward down arrows. This indicated the GDP had caused the FDI and the correlation was negative. This observation is consistent in the 2018–2019 period. However, upon reaching the year 2020, the pattern reshifted towards a positive correlation where GDP led FDI. In 2016 and 2020, in the short-term (high frequency) indicated positive and negative correlations where FDI had caused GDP, respectively. The large area of blue colour confirms the findings for the causality analysis to reflect a non-directional causality in the African region, as presented in Table 6.

**Fig 5:**
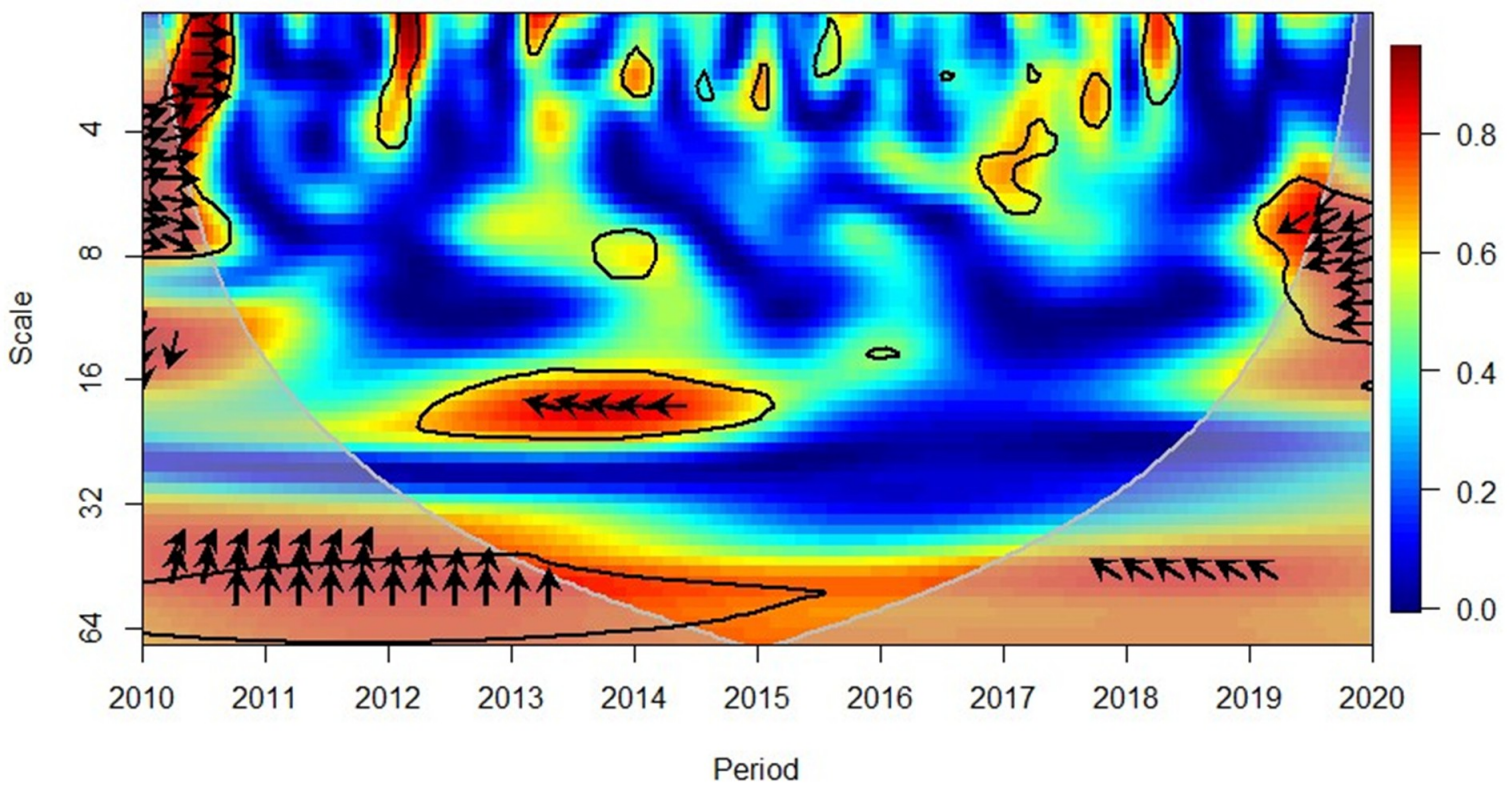
America region, GDP and FDI. Source: Authors’ compilation using R-Software.

Fig 5 depicts that in mid-2010, the American region reflected a positive correlation in the short-term (high frequency) between GDP and FDI. The rightward down arrows indicated that GDP leads FDI. However, the medium-term (medium frequency) of 2013–2015 relationship was negative, and the leftward up arrows indicate that FDI caused GDP growth. Yet, in mid-2019, it was again observed through the downward and left arrows that the shift changed to GDP leading FDI, and negative correlation was consistent.

**Fig 6:**
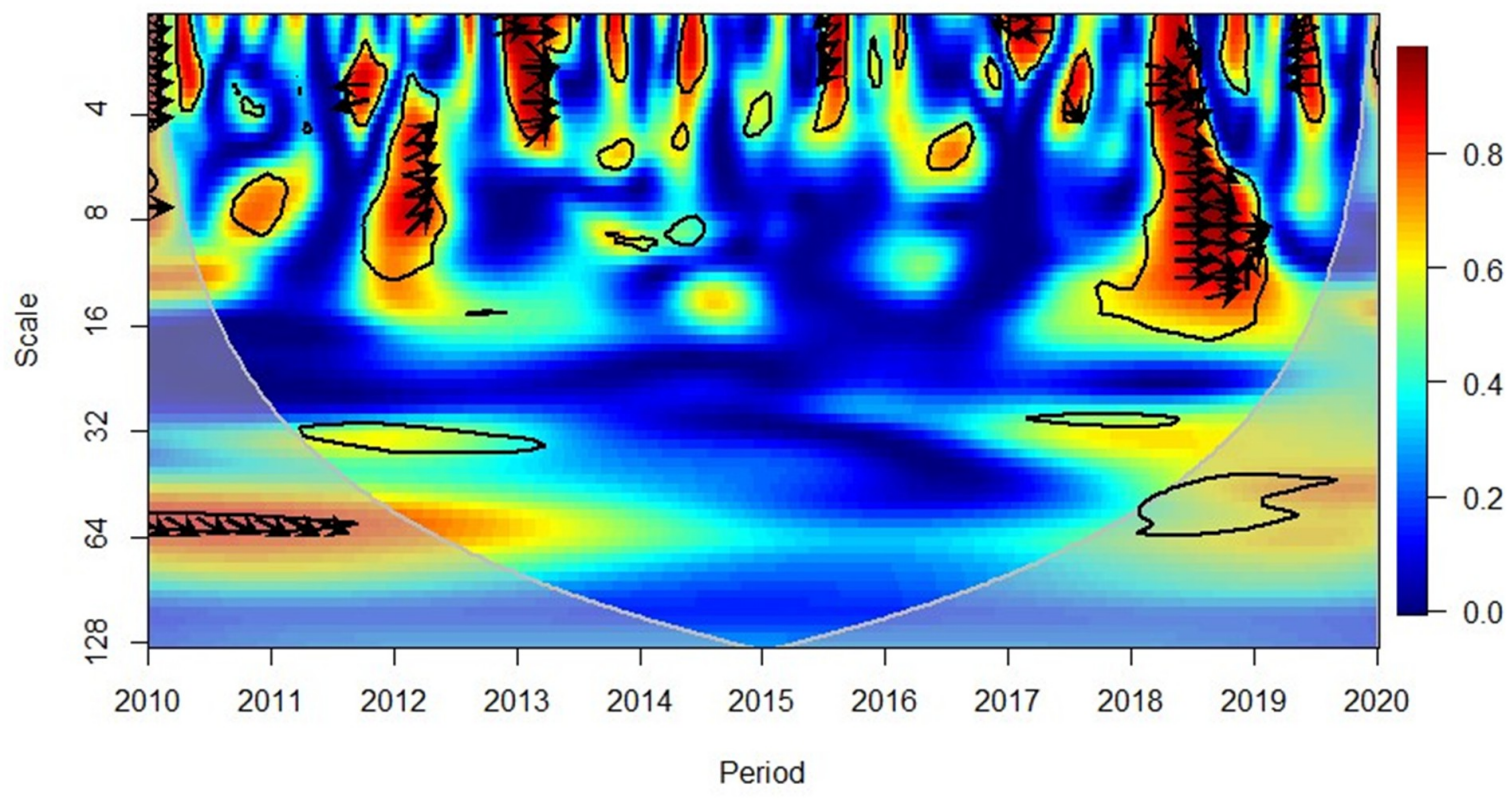
Asian region, GDP and FDI. Source: Authors’ compilation using R-Software.

Fig 6 indicates that from 2010 to 2013, the Asian region indicated a positive relationship between GDP and FDI except for mid-2011 (where the relationship was negative, and GDP has led FDI in the short-term (high frequency). Rightward and up arrows indicated that FDI caused GDP in this period 2015–2017 reflected a negative correlation between the variables and the leftward and down arrows (at short-term high frequency) depicted that GDP led FDI; this changed the positive relationship in mid-2017. The 2018–2019 period again reflected a positive correlation in short to medium-term (high to medium frequencies) and rightward and up arrows indicated that the FDI had led GDP. However, the relationship between the variables was again reflected to be negative in mid-2019 and at a short-term (high frequency), it has reflected that GDP leads FDI. These results reach conformity to the causality analysis results depicted bi-directional interaction between the variables (Table 6).

**Fig 7:**
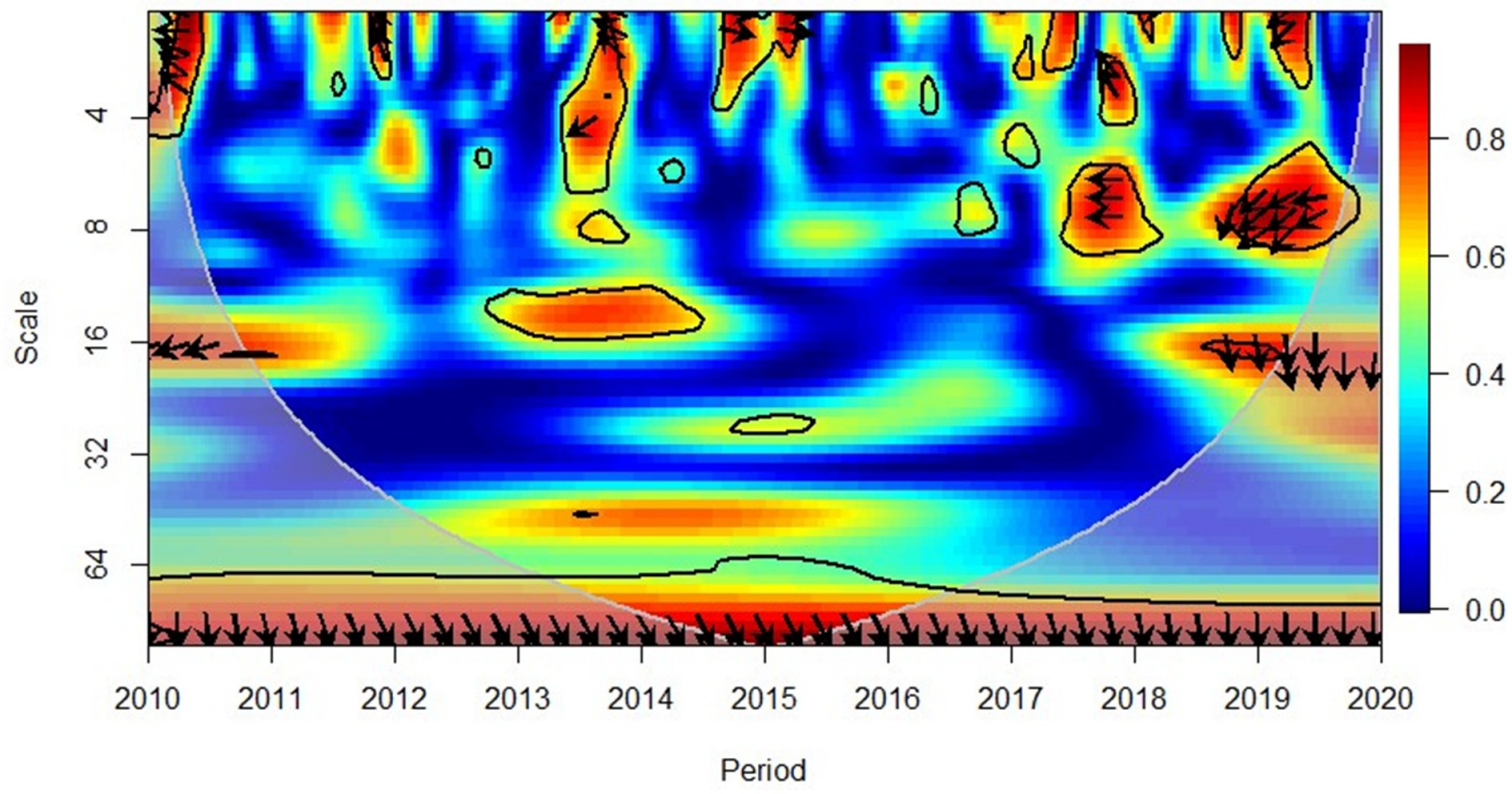
European region, GDP and FDI. Source: Authors’ compilation using R-Software.

The region of Europe indicates (Fig 7) a rather complex picture in terms of the GDP and FDI. The 2010–2012 period indicates a negative relationship between the variables in the short-term (high frequency) and the leftward and up arrows indicate FDI led GDP. Mixed results were observed in 2014, where the negative correlation was indicated, yet the causality goes both ways. 2015 was a year that reflected a positive correlation between GDP and FDI (long term, low frequency). The rightward and up arrows indicated that GDP led FDI. In 2018, it also reflected observations similar to those in 2014 and 2019, indicating that GDP leads FDI at short to medium-term (medium to high frequencies). This is probably attributable to the range of nations comprised in the European region and their varied investment patterns throughout the world.

**Fig 8:**
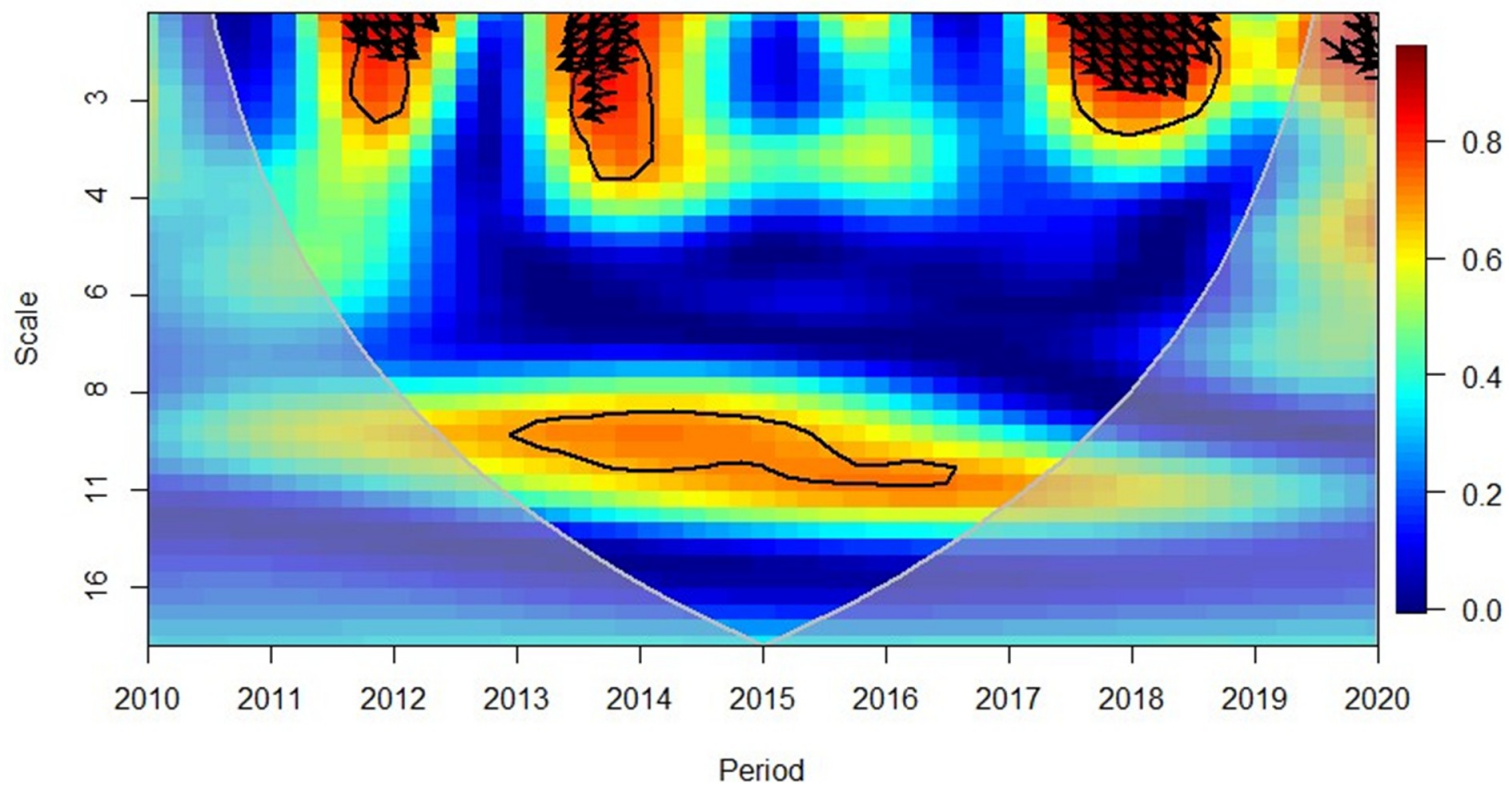
Mediterranean region, GDP and FDI. Source: Authors’ compilation using R-Software.

Concerning the Mediterranean region (Fig 8) in 2012, a positive correlation was observed between GDP and FDI. The rightward and down arrows reflected in the short-term (high frequency) indicated that GDP caused FDI. However, the correlation between the variables in 2013–2014 was negative and in the short-term (high frequency), and the leftward and down arrowheads indicated that GDP had caused FDI. 2018–2019 period again stays consistent with the observations similar to 2012, justifying the findings of the causality analysis in Table 6. Thus, no clear directional causality was observed throughout the period from 2010 to 2020.

**Fig 9:**
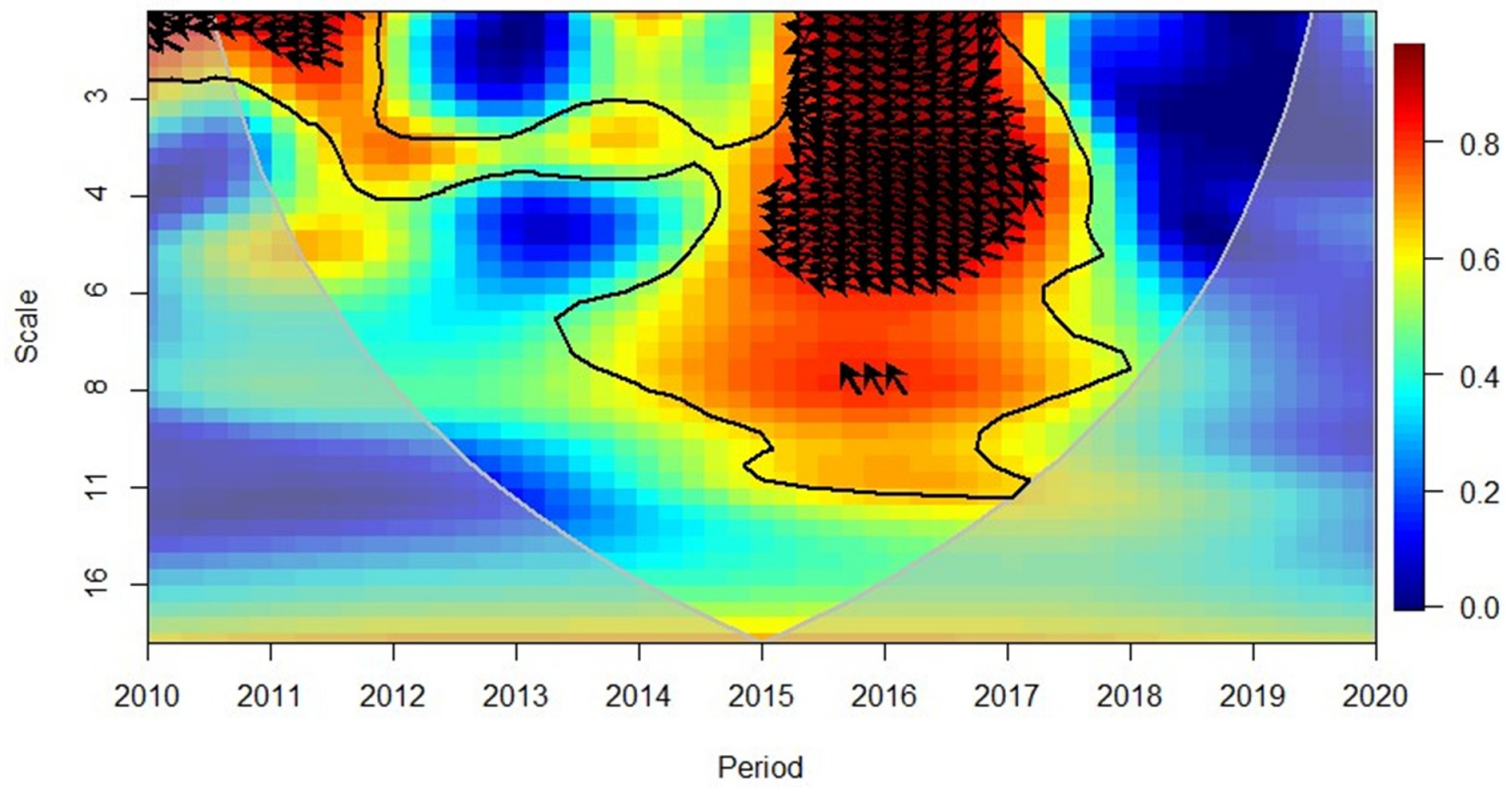
Oceania region, GDP and FDI. Source: Authors’ compilation using R-Software.

The period of 2011 to 2012 reflected a negative correlation between GDP and FDI in the Oceania region (Fig 9), and in the short-term (high frequency), the leftward and up arrows indicated that FDI has led GDP. Forthe period 2015–2018, the correlation was still observed to be negative. In the short to medium-term (high and medium frequencies), and the arrowheads directed both leftward and up (FDI leads GDP) as well as leftward and down (GDP leads FDI), further bringing conformity to the causality analysis findings in Table 6.

### DH Granger non-causality testing approach

For the study, the Granger causality test for heterogeneous non-causality proposed by Dumitrescu and Hurlin [99] was utilised to investigate the causal relationship among the panel series. The causality at the first difference is examined from GDP to FDI, and vice versa. The results of the Granger causality test are shown in Table 4.

**Table 4:** Dumitrescu and Hurlin panel Granger causality results.

Region	Test	Causality from DGDP to DFDI	Causality from DFDI to DGDP
Global	HENC	2.8200***	3.5628***
African	HENC	0.6954	-0.0113
American	HENC	2.3124**	0.7625
Asian	HENC	2.9267***	5.1259***
European	HENC	-0.058	0.4814
Mediterranean	HENC	0.2078	-0.0784
Oceanian	HENC	-0.0969	1.4079

Note: HENC: Heterogeneous Non-Causality Hypothesis

**Reject H_0_ at a 5% level of significance;

***Reject H_0_ at a 1% level of significance

Source: Authors’ calculations

The bi-directional causality exists between FDI and GDP per capita in the global context at a 1% significance level. Results of the region-wise analysis show bi-directional causality in the Asian region at a 1% significance level, indicating that FDI has substantial predictive power on the economic growth of the Asian countries examined. A uni-directional causality from GDP to FDI exists in the American region at the significance level of 5%. The test findings also indicate no evidence of causality between GDP and FDI in African, European, Mediterranean, or Oceanian regions at 1%, 5%, and 10% levels of significance, respectively, concerning the HENC hypotheses. Moreover, the global analysis and the Asian region confirm the FDI-led growth hypothesis and growth-led FDI hypothesis, while the American region only corroborates the growth-led FDI hypothesis.

Table 5 illustrates the results of Block Exogeneity Wald test for the sample. Two panel Granger causality tests were conducted namely, the simple bivariate test and the block exogeneity test–to provide some preliminary results on the interdependency between the FDI and GDP. Results of Block Exogeneity Wald test further confirm panel Granger causality test results in Table 4.

**Table 5:** Panel VAR/block exogeneity test results.

	Global	African	American	Asian	European	Mediterranean	Oceanian
GDP does not cause FDI	0.388***	0.864	5.414**	1.332*	2.056	0.923	0.348
FDI does not cause GDP	0.912**	1.736	0.665	1.535**	0.769	0.013	0.052

Note:

*Reject at a 10% level of significance;

**Reject at a 5% level of significance;

***Reject at a 1% level of significance

Source: Authors’ calculations

Tables 6 summarises the results of the Granger-causality analysis and wavelet coherence for GDP-FDI and FDI-GDP. The wavelet coherence technique captures the time dependence of the variables which is conjointly captured under the Granger causality approach. Accordingly, the findings revealed that despite the slight inconsistencies noted, the overall finding of both techniques brings unanimous results, bringing justifications to the study.

**Table 6:** FDI-economic growth causality summary.

Region	Test	GDP-FDI	Causality findings	Wavelet coherence findings
Global	HENC	Bi-directional causality	GDP ↔ FDI	-
African	HENC	Non-directional causality	GDP ⇹ FDI	No relationships throughout the period.
American	HENC	Uni-directional causality	GDP → FDI	GDP led FDI in 2010.
Asian	HENC	Bi-directional causality	GDP ↔ FDI	Bi-directional relationships are observed.
European	HENC	Non-directional causality	GDP ⇹ FDI	Minimal mixed relationships are observed.
Mediterranean	HENC	Non-directional causality	GDP ⇹ FDI	Mixed relationships are observed.
Oceanian	HENC	Non-directional causality	GDP ⇹ FDI	No clear relationships after 2018.

Note: HENC: Heterogeneous Non-Causality Hypothesis

Source: Authors’ calculations

The results of the S3 Appendix can be summarised as follows; no causality is observed in the emerging and frontier panels of Africa, an emerging panel of America, developed and emerging panels of Europe, emerging and frontier panels of the Mediterranean region and the developed panel of the Oceanian region. There is a bidirectional causality in the frontier panel of Asia at a 1% significance level. Considering uni-directional causality, America and Asia developed panels show causality from GDP to FDI at 1% and 10% significance levels, respectively. Apart from this, the emerging Asian panel and the European frontier panel reveal causality from FDI to GDP at 1% and 10% levels, respectively.

The European Sovereign Debt Crisis was considered for Global and European regional analysis. The findings revealed no significant impact from the recession on either GDP per capita or net FDI inflows. The results are reported in the S4 Appendix.

Foreign Direct Investment is often regarded as having a significant impact on economic growth. In other words, FDI is an economic stimulant, particularly in developing, emerging, and impoverished countries. Here, FDI facilitates technology transfer from developed to developing, emerging, and underprivileged countries. It enables domestic investment and institutional improvement and human capital development in the host countries. Similarly, economic growth encourages and supports FDI. The reported bi-directional result for the Asian region is consistent with Liu, Shu [49] and Mahmoodi and Mahmoodi [24].

The bi-directionality between economic growth and FDI in Asia could be mainly due to foreign investment accelerating economic growth and exposure to new technology, creation of job opportunities, in addition to other reasons. In return, increased economic growth can attract more FDI, resulting in a cyclical relationship between the two variables.

The uni-directional causality from GDP to FDI in the American region probably be caused by many of the Latin American developing countries attracting FDI due to economic growth. Another reason is the foreign investors’ view that investments in the Latin American region may produce greater returns in the future.

Results obtained for Africa were also substantiated by Mahembe and Odhiambo [10] for low-income SADC countries. This could be due to low levels of economic growth in this region, which deter foreign investors. This scenario, in turn, can make the causality between economic growth and FDI insignificant. It could also be assumed that the uni-directional causality between the MICs in SADC observed by Mahembe and Odhiambo [10] is offset by most LICs in the entire African region.

The results for the Mediterranean region are consistent with the study by Asheghian [72], according to which several political events, such as the regime change in 1979 and the Iran-Iraq war (1980–1988), have deterred Iran’s conductivity to FDI over time. This could also be applied to the entire Mediterranean region for the period considered. The reason was eruptions of several prominent political conflicts and wars in the region from 2010 onwards, such as the Yemeni al-Qaeda crackdown (2010–2015), the intensification of proxy conflict between Saudi Arabia and Iran (2011), the Syrian civil war (2011-present), Yemeni crisis (2011-present), Egyptian crisis (2011–2014), Syrian civil war spillover in Lebanon (2011–2017), the war in Iraq (2013–2017) and Iraqi insurgency (2017-present). These can be observed through the significant decline in mean FDI inflows in 2011 and the overall downward trend until 2015. These conflicts may have caused foreign investors to view the Mediterranean region as an unfavourable destination for FDI, thus rendering each variable incapable of significantly causing the other. However, the impact of each of these political events on FDI inflows into the region requires further investigating using advanced root cause analysis techniques.

Non-directionality of the variables in Oceania is consistent with the findings of the study by Pandya and Sisombat [75], in which it is stated that FDI’s role in an economic expansion is questionable in developed countries such as Australia. The Organisation for Economic Co-operation and Development (OECD) index for FDI restrictiveness places Oceania well above the OECD average. Hence, Oceanian’s foreign investment policy is likely to have a material effect on the non-causality between FDI and economic growth in the region.

Golitsis, Avdiu [67] and Nath [66] testify to the non-directionality between economic growth and FDI found in Europe. The cause behind the non-causality between the variables in Europe and Oceania could stem from the fact that major economies in these regions are developed countries, which are investors as opposed to destinations of FDI. Since only the FDI inflows have been taken into account, it do not exert influence on economic growth or vice versa in these regions, which implies a limitation of the present study. However, the reasons why Europe and Oceania show a non-directional causality while the American region shows a uni-directional causality are unclear. This should be subjected to additional analysis in future research.

## Concluding remarks

This study intended to assess the causal nexus between FDI and economic growth for 117 selected countries during the 2010–2020 period, using a bivariate panel Granger causality analysis, panel VAR/block exogeneity test and Wavelet Coherence approach, under two classifications; region-wise globally, and region-wise cross-markets as developed, emerging and frontier markets. The research focused on answering one crucial question: does FDI spur economic growth or vice versa?. To measure the dynamic causal relationship between the variables, the study deployed a heterogeneous panel non-causality technique over the other causality techniques. According to empirical findings, the Granger causality analysis for the global context revealed bi-directional causality between FDI and economic growth. The Asian region indicated that if FDI inflows are effectively channelled, economic growth could be improved. In contrast, it was found that a uni-directional causality was present from economic growth to FDI in the American region. The results also confirmed a non-directional causality in the European, Oceanian, Mediterranean, and African regions. Therefore, it can be surmised that FDI inflows contribute to the economy and vice versa. Hence governments should continue to adopt policies that promote a favourable investment climate to attract FDI. The strong tendency of either no Granger-causality or Granger-causality between GDP and FDI suggests that FDI could play a significant role in propelling the economies of these investigated regions. Moreover, the wavelet coherence approach validated the results, enabling the exploration of short-run and long-run causal linkages used to analyse the co-movement between FDI and economic growth.

This research identified several policy implications for the host economy to realise the benefits of FDI inflows. The host countries should devise policies that encourage FDI targeting high-growth and priority sectors of the host economy. The host economy should establish these absorptive properties in terms of infrastructure, financial markets, human capital base, market size, and economic and political stability. Identifying these prerequisites and regular review of these in the host country will influence how FDI can reap benefits. Therefore, minimising the potential risks is crucial. In addition, it is advised that the government of both developed and emerging economies should emphasise their attention on encouraging domestic and foreign investment for regional growth.

In addition, the host economy should implement reforms to lower barriers, address loopholes in the existing structure and establish a favourable environment that attracts and retains FDI. Sustainability of this conducive environment and revising of policies to adapt to changes in the global environment are also vital.

Future studies can expand the analysis by examining more FDI determinants with a sectoral analysis that can reveal the differences across the sectors. Moreover, additional studies can distinguish the causality under various economic conditions (recessions and expansions).

## Supporting information

S1 Appendix Summary of related literature. (DOCX)

S2 Appendix Data file. (XLSX)

S3 Appendix Results of Granger causality test for cross-markets for the countries in each region. (DOCX)

S4 Appendix Results of panel regression for global and European region with European sovereign debt crisis as a recession. (DOCX)
