# Supplementary material for: More than just investment: Causality analysis between foreign direct investment and economic growth
Source: PLoS One. 2022 Nov 3;17(11):e0276621. doi: 10.1371/journal.pone.0276621 (PMC9632898; doi:10.1371/journal.pone.0276621)
Supplement: S1 Appendix — (DOCX) [file pone.0276621.s001.docx]

**S1 Appendix: Summary of related literature**

**S1.1: Global**

| **Authors** | **Year** | **Sample Country** | **Time Period** | **Econometric Technique** | **Major Findings** |
| --- | --- | --- | --- | --- | --- |
| Rudra P. Pradhan,  Mak B. Arvin, John H. Hall & Mahendhiran Nair | 2016 | 19 Eurozone countries | 1988–2013 | Vector Error Correction Model | FDI, financial development, and trade openness show a two-way Granger causality with per capita economic growth , and financial development and trade openness show a two-way causality in the short run. There is a one-way causality from per capita economic growth to trade openness. |
| Majid Mahmoodi & Elahe Mahmoodi | 2016 | 8 European developing countries and 8 Asian developing countries | 1986 - 2013 | Panel Vector Error Correction Model causality | In the short-run, there is a two-way causality between GDP and FDI, a unidirectional causality from GDP and FDI to exports in European there is bidirectional causality between exports and economic growth in the short-run |
| Fayyaz Ahmad,  Muhammad Umar Drazb & Su-Chang Yang | 2018 | ASEAN Countries | 1981-2013 | Unit root testing and co-integration and  causality tests | Long and short-run causality running from exports and FDI to economic growth |
| Umer Jeelanie Banday,  Saravanan Murugan &  Javeria Maryam | 2020 | BRICS countries | Time series data 1990–2018 | Autoregressive Distributed Lag model Granger causality tests | Bi-directional causality from FDI to economic growth. |
| Samir Saidi, Venkatesh Mani, Haifa Mefteh, Muhammad Shahbaz & Pervaiz Akhtar | 2020 | 46 developing countries | Global panel data 2000–2016 | Generalized Method of Moments approach | Bi-directional relationship between FDI economic growth and transport infrastructure in all panels. |
| Sasi Iamsiraroj &  Mehmet Ali Ulubaşoğlu | 2015 | 140 Countries | 1970 - 2009 |  | FDI positively affects economic growth |
| Nicholas Apergis, Katerina Lyroudi & Athanasios Vamvakidis | 2008 | 27 economies in transition | 1991 - 2004 | Panels cointegration test, Pooled Mean Group estimator | FDI has a signiﬁcant relationship with economic growth in transition countries with high levels of income |
| Wu Jyun-Yi & Hsu Chih-Chiang | 2008 | 62 Countries | 1975 - 2000 | Threshold regression techniques | FDI is found to have a positive and significant impact on growth when host countries have better levels of initial GDP and human capital |
| Mousumi Duttaray, Amitava K. Dutt, & Kajal Mukhopadhyay | 2008 | 66 developing countries | 1970-1996 | Granger causality test | FDI causes growth in several of the developing countries through different mechanisms, and reverse causality from growth to FDI exists for many countries |
| Sahraoui Mohammed Abbes, Belmokaddem Mostéfaa, Guellil Mohammed Seghir & Ghouali Yassine Zakarya | 2015 | 65 countries | 1980 – 2010 | Co-integration and panel Granger causality tests | Unidirectional causality from FDI to GDP |
| Alper Aslan, Buket Altinoz & Melike Atay Polat | 2021 | N-11 countries | 1980 - 2018 | Panel Vector Autoregression model | Unidirectional causalities from carbon dioxide emissions to GDP, from energy consumption to GDP, from FDI to Carbon dioxide emissions and GDP, from financial development to GDP |
| Priya Gupta and Archana Singh | 2016 | BRICS nations | 1992 - 2013 | Granger causality test | Uni-directional causality running from GDP to FDI in the long run |

**S1.2: African region**

| **Authors** | **Year** | **Sample Country** | **Time Period** | **Econometric Technique** | **Major Findings** |
| --- | --- | --- | --- | --- | --- |
| Erasmus L. Owusu | 2020 | Namibia | 1990 - 2016 | Multivariate Granger-causality within an Auto-regression distributed lag-bounds approach,  Co-integration and unrestricted error correction model | Strong bi-directional causal relationship between FDI inflow and economic growth both in the short and the long run |
| Ada Chigozie Akadiri, Hasan Gungor, Seyi Saint Akadiri &  Mojibola Bamidele-Sadiq | 2019 | 25 African countries | 1980-2018 | Panel bootstrapping cointegration techniques, Granger causality test | Bidirectional causality relationship between FDI and economic growth and between FDI and trade. The trade-led growth, FDI-led growth, growth-led trade, and growth-led FDI hypotheses were confirmed |
| Edmore E. Mahembe and Nicholas M. Odhiambo | 2016 | SADC countries | 1980-2012 | Panel-data analysis  methods | Uni-directional causality from GDP to FDI in middle-income countries. there is no causality in either direction in low-income countries |
| Tafirenyika Sunde | 2017 | South Africa | 1970-2012 | Autoregressive Distributed Lag bound testing approach, Vector Error Correction Model Granger causality approach | Uni-directional causality running from FDI to economic growth. |
| [Nicholas M. Odhiambo](#_ENREF_45)  [(2021)](#_ENREF_45) | 2021 | Kenya | Secondary Data 1980–2018 | Autoregressive Distributed Lag bounds testing approach, Multivariate Granger Causality | Unidirectional causal flow from economic growth to FDI in long-run and short-run |
| Rifat Baris Tekin | 2012 | African Least developed  countries | 1970-2010 | Panel-data  approach | No significant causality relation among foreign aid, openness to trade, and economic growth |
| M. Belloumi | 2014 | Tunisia | 1970–2008 | Autoregressive Distributed Lag Bounds testing approach to cointegration  and Granger causality test | No significant Granger causality from FDI to economic growth, from economic growth to FDI. |

**S1.3: American region**

| **Authors** | **Year** | **Sample Country** | **Time Period** | **Econometric Technique** | **Major Findings** |
| --- | --- | --- | --- | --- | --- |
| Omar M. Al Nasser | 2010 | 14 Latin American and Asian countries | 1978-2003 | Panel data modelling technique | One-way causality from economic growth to FDI for Asian countries and a Bi-directional causality for Latin American countries |
| Victor Owusu-Nantwi & Christopher Erickson | 2019 | South America | 1980–2015. | Pedroni’s cointegration test, Vector Error Correction Model | Bidirectional causality between FDI and economic growth in short run |
| Olugbenga Onafowora & Oluwole Owoye | 2019 | 5 Caribbean countries | 1975–2015 | Granger causality test | Bidirectional causality between economic growth and FDI in the Bahamas. Unidirectional causality from output growth to FDI in Barbados and Dominican Republic; from FDI to economic growth in Jamaica and Trinidad and Tobago and Unidirectional causality from output growth to FDI in Barbados and Dominican Republic, from FDI to economic growth in Jamaica and Trinidad and Tobago and a bidirectional causality in the Bahamas |
| Rania Ihab Naguib | 2012 | Argentina | 1971–2000 | Error Correction model | FDI have no effect on either short- or long-run economic growth in Argentina. |
| Chen-Chang Lo, Yaling Lin, Tsung-Li Chi & Dominique Jude Joseph | 2013 | Haiti | 1980-2010 | System of two simultaneous equations has been estimated by the two-stage least squares | Economic growth has no significant impact on FDI and vice versa |

**S1.4: Asian region**

| **Authors** | **Year** | **Sample Country** | **Time Period** | **Econometric Technique** | **Major Findings** |
| --- | --- | --- | --- | --- | --- |
| Palamalai Srinivasan, M. Kalaivani, & P. Ibrahim | 2011 | SAARC countries | 1970-2007 | Johansen’s cointegration test, Vector Error Correction Model | Bidirectional causal link between GDP and FDI for the selected nations except India, where there is a one-way long-run causality from GDP to FDI in long-run |
| Xiaohui Liu , Peter Burridge  & P. J. N. Sinclair | 2002 | China | 1981:1 - 1997:4 | Vector Autoregression- Error Correction Model framework | Two-way causal connections exist between  economic growth, FDI |
| N. Balamurali & C. Bogahawatte | 2004 | Sri Lanka | 1977-2003 | Granger Causality Tests from Error Correction Model | Bidirectional causality between FDI and economic growth |
| Manpreet Kaur, Surendra S.  Yadav, & Vinayshil Gautam | 2013 | India | post liberalization period, i.e., post 1991 pre-liberalization period, i.e., pre-1991 | Granger Causality | Bidirectional causality between FDI and growth in post liberalization period. FDI-led growth is seen in pre liberalization period |
| Sajid Anwar & Lan Phi Nguyen | 2010 | 61 provinces of Vietnam | 1996–2005 | Generalized Method of Moments estimation | Mutually reinforcing two-way linkage between FDI and economic growth |
| Muhammad Shahzad Iqbal, Faiz Muhammad Shaikh & Amir Hussain Shar | 2010 | Pakistan | 1998 - 2009 | Vector Error Correction Model causality test | Bidirectional causality between FDI, exports and economic growth |
| [Abbas Ali Chandio, Amir Ali Mirani *&*  Rashid Usman](#_ENREF_14) Shar | 2019 | Pakistan | 1991 - 2013 | Vector Error Correction Model Granger causality test Auto Regressive Distributed Lag approach | Bidirectional causality between economic growth and agricultural sector FDI in both short and long run |
| Xiaohui Liu , Chang Shu & Peter Sinclair | 2009 | 9 Asian Countries | 1970 - 2002 | Vector Error Correction Model framework | Two-way causal connections between trade, inward FDI, and growth for most of the sample economies |
| Changwen Zhao & Jiang Du | 2014 | China | **-** | Vector Autoregression model | Non-significant two-way causality between FDI and growth |
| Ranjan Kumar Dash & Chandan Sharma | 2011 | India | 1991Q3 - 2006Q3 | Vector Autoregression model, Ganger non-causality test | Bidirectional causality between FDI and economic growth |
| Abdur Chowdhury and George Mavrotas | 2005 | 3 developing countries, namely Chile, Malaysia and Thailand | 1969-2000 | Toda-Yamamoto test for causality | GDP causes FDI in the case of Chile and not vice versa. For both Malaysia and Thailand, there is a strong bi-directional causality between the two variables |
| Frank S.T. Hsiao and Mei-Chu W. Hsiao | 2006 | 8 rapidly developing East and Southeast Asian economies | 1986 - 2004 | Granger causality test | Unidirectional causality running from FDI to GDP |
| G.Jayachandran & A. Seilan | 2010 | India | 1970-2007 | Cointegration analysis Granger causality test | Unidirectional causality from FDI to growth rate |
| Soo Khoon Goh, Chung Yan Sam & Robert McNown. | 2017 | 11 Asian countries | 1970-2012 | Newly developed cointegration test, the bootstrap Autoregressive Distributed Lag | Granger causality running from FDI to GDP in Hong Kong, Japan, Korea, Malaysia, the Philippines, and Singapore in short run |
| Mete Feridun & Yaya Sissoko | 2011 | Singapore | 1976 – 2002 | Granger causality and Vector Auto Regression | Unidirectional causality from FDI to economic growth |
| Bibhuti Sarker & Farid Khan | 2020 | Bangladesh | 1972 - 2017 | Unit root tests Augmented Autoregressive Distributed Lag modeling approach Granger causality test | Unidirectional causality running from GDP to FDI |
| Ergin Akalpler & Hemn Adil | 2017 | Singapore | 1980-2014 | Vector Error Correction Model | Strong evidence of the absence of a long-run relationship or causality that runs from gross savings, FDI, trade and gross fixed capital formation to economic growth. |
| Seng Sothan and Xibin Zhang | 2017 | Cambodia | 1980–2014 | Granger causality  test based on the vector error  correction model | No causality that run from GDP to FDI |

**S1.5: European region**

| **Authors** | **Year** | **Sample Country** | **Time Period** | **Econometric Technique** | **Major Findings** |
| --- | --- | --- | --- | --- | --- |
| Argiro Moudatsou | 2003 | European Union (EU) countries | 1980-1996 | Regression analysis | FDI inflows have a significant effect on growth |
| Melina Dritsaki,  Chaido Dritsaki & Antonios Adamopoulos | 2004 | Greece | 1960-2002 | Cointegration analysis Granger causality test Error correction model | Unidirectional causality from FDI to GDP |
| Krešimir Čičak & Petar Sorić | 2015 | European Transition Countries |  | Granger causality test | FDI has a positive impact on GDP growth rate in most of the analyzed countries, especially, in Poland, Czech Republic and Hungary. |
| Hossein Varamini & Svetlana Kalash | 2010 | 10 emerging economies in Europe | 1994 - 2003 | Granger causality test | GDP growth has a unilateral Granger-cause on FDI inflows for 9 of 10 emerging European economies No Granger causality from FDI inflows to the changes in economic growth of any of the 10 countries |
| Kuo Cheng Kuo, Sue Ling Lai, Khunlaphat Chancham & Ming Liu | 2014 | Germany | 1971-2010 | Granger causality | Unidirectional causality running from GDP to energy consumption and from GDP to FDI |
| Sam Hobbs , Dimitrios Paparas & Mostafa E. AboElsoud | 2021 | Albania | 1992-2016 | A general macroeconomic model | Unidirectional causality from economic growth to exports and FDI in the short term but not vice versa |
| Hiranya K. Nath | 2009 | 13 transitions economies of  Central and Eastern Europe, and  the Baltic region. | 1991 -2005 | Panel data estimation techniques | FDI does not have any significant impact on growth |
| Petros Golitsis,  Kushtrim Avdiu &  Leslie T. Szamosi | 2018 | Albania with  76 observations. | 1996 - 2014, using quarterly data | Vector Error  Correction model | No relationship between FDI, economic growth, and capital formation. |

**S1.6: Mediterranean region**

| **Authors** | **Year** | **Sample Country** | **Time Period** | **Econometric Technique** | **Major Findings** |
| --- | --- | --- | --- | --- | --- |
| Ihsan Gunaydin & Ekrem Tatoglu | 2005 | Turkey | 1968‐2002 | Cointegration, Error‐Correction models and the Augmented Vector Autoregressive methodology | Bidirectional Granger causality between FDI and economic growth |
| Soheila Khoshnevis Yazdi, Khadijeh Homa Salehi & Mahshid Soheilzad | 2015 | Iran | 1985–2013 | Autoregressive Distributed Lag bounds testing of cointegration and Granger causality analysis | Bidirectional causalities for FDI, real effective exchange rate, real GDP and tourism expenditure and unidirectional relationship from real effective exchange rate, real GDP and tourism expenditure to FDI. |
| Maha Kalai & Nahed  Zghidi | 2017 | 15 selected Middle Eastern and North African countries | 1999–2012 | Co-integration and Vector Error Correction Model | Unidirectional relationship running from FDI to economic growth in long-run |
| Atef Saad Alshehry | 2015 | Saudi Arabia | 1970-2012 | Granger causality tests | FDI inflows promote both short- and long-term economic growth. |
| Dalia M. Ibrahiem | 2015 | Egypt | 1980 - 2011 | Auto Regressive Distributed Lag bound testing approach Granger causality test | Unidirectional causality running from FDI to economic growth |
| Parviz Asheghian | 2016 | Iran | 1971–2007 | Granger non-causality test | No causal relationship between FDI growth and GDP per capita growth in either direction |

**S1.7: Oceanian region**

| **Authors** | **Year** | **Sample Country** | **Time Period** | **Econometric Technique** | **Major Findings** |
| --- | --- | --- | --- | --- | --- |
| T.K. Jayaraman & Chee-Keong Choong | 2006 | Fiji | 1970–2001 | Granger non-causality test based on Vector Error Correction Model | Bidirectional causality between FDI and economic growth |
| Krishna G. Iyer , Alicia N. Rambaldi & Kam Ki Tang | 2009 | Australia | Quarterly data  1988 - 2003 | Cointegrated Vector Autoregressive model | All three types of foreign investment Granger cause GDP in the short run. FDI has a long run forcing effect on GDP |
| Viral Pandya & Sommala Sisombat | 2017 | Australia | 2001 - 2013 | Multiple regression | No relationship between FDI and economic growth of Australia |
