# Supplementary material for: More than just investment: Causality analysis between foreign direct investment and economic growth
Source: PLoS One. 2022 Nov 3;17(11):e0276621. doi: 10.1371/journal.pone.0276621 (PMC9632898; doi:10.1371/journal.pone.0276621)
Supplement: S3 Appendix — (DOCX) [file pone.0276621.s003.docx]

**S3 Appendix: Results of Granger causality test for cross-markets for the countries in each region**

| **Region** | **GDP to FDI** | **FDI to GDP** | **Causality Findings** |
| --- | --- | --- | --- |
| **African** |  |  |  |
| **Emerging** | **0.0243** | **-0.6356** | **GDP ⇹ FDI** |
| South Africa | 0.0243 | -0.6356 | GDP ⇹ FDI |
| **Frontier** | **-1.4170** | **-0.2252** | **GDP ⇹ FDI** |
| Kenya | -0.6830 | 2.9614*** | FDI → GDP |
| Mali | -0.7004 | -0.4056 | GDP ⇹ FDI |
| Morocco | -0.3467 | -0.6080 | GDP ⇹ FDI |
| Nigeria | -0.2658 | -0.6872 | GDP ⇹ FDI |
| Senegal | -0.6810 | -0.5575 | GDP ⇹ FDI |
| Togo | -0.6899 | -0.7070 | GDP ⇹ FDI |
| Tunisia | -0.3822 | -0.5920 | GDP ⇹ FDI |
| **American** |  |  |  |
| **Developed** | **3.0371***** | **0.9452** | **GDP → FDI** |
| Canada | 4.7873*** | 0.0514 | GDP → FDI |
| United States | -0.4923 | 1.2853 | GDP ⇹ FDI |
| **Emerging** | **-0.3005** | **-0.9673** | **GDP ⇹ FDI** |
| Brazil | -0.6416 | -0.7067 | GDP ⇹ FDI |
| Chile | 0.7175 | -0.4957 | GDP ⇹ FDI |
| Colombia | -0.5293 | -0.5372 | GDP ⇹ FDI |
| Mexico | 0.4685 | 0.2003 | GDP ⇹ FDI |
| Peru | -0.6871 | -0.6236 | GDP ⇹ FDI |
| **Asian** |  |  |  |
| **Developed** | **1.7173*** | **-0.6758** | **GDP → FDI** |
| Hong Kong | 3.8738*** | -0.7030 | GDP → FDI |
| Israel | -0.0036 | 0.2823 | GDP ⇹ FDI |
| Japan | -0.2955 | -0.2266 | GDP ⇹ FDI |
| Singapore | -0.1401 | -0.7045 | GDP ⇹ FDI |
| **Emerging** | **0.6832** | **3.1520***** | **FDI → GDP** |
| China | -0.5858 | -0.2145 | GDP ⇹ FDI |
| India | -0.6274 | -0.6963 | GDP ⇹ FDI |
| Indonesia | 0.7000 | 1.2551 | GDP ⇹ FDI |
| Korea, Rep. | 2.6496*** | 11.2959*** | GDP ↔ FDI |
| Kuwait | -0.5488 | -0.2158 | GDP ⇹ FDI |
| Malaysia | 3.0199*** | -0.6812 | GDP → FDI |
| Philippines | -0.6911 | -0.0255 | GDP ⇹ FDI |
| Qatar | -0.3650 | -0.1901 | GDP ⇹ FDI |
| Thailand | -0.6951 | -0.5106 | GDP ⇹ FDI |
| Turkey | -0.6959 | -0.0495 | GDP ⇹ FDI |
| **Frontier** | **3.6261***** | **2.9598***** | **GDP ↔ FDI** |
| Bangladesh | 8.3419*** | 0.3230 | GDP → FDI |
| Pakistan | -0.2435 | 6.6636*** | FDI → GDP |
| Sri Lanka | -0.6257 | -0.3827 | GDP ⇹ FDI |
| Vietnam | -0.2204 | -0.6844 | GDP ⇹ FDI |
| **European** |  |  |  |
| **Developed** | **0.6982** | **-1.0585** | **GDP ⇹ FDI** |
| Austria | 0.7540 | -0.7020 | GDP ⇹ FDI |
| Belgium | 0.6722 | 0.1257 | GDP ⇹ FDI |
| Denmark | -0.4725 | -0.1800 | GDP ⇹ FDI |
| Finland | -0.4040 | -0.5246 | GDP ⇹ FDI |
| France | -0.6849 | -0.4770 | GDP ⇹ FDI |
| Germany | -0.2551 | 0.1525 | GDP ⇹ FDI |
| Ireland | -0.0948 | 0.2929 | GDP ⇹ FDI |
| Italy | -0.6648 | -0.6555 | GDP ⇹ FDI |
| Netherlands | -0.0711 | -0.7065 | GDP ⇹ FDI |
| Norway | 2.0566** | -0.7054 | GDP → FDI |
| Portugal | -0.7071 | -0.4028 | GDP ⇹ FDI |
| Spain | 3.5282*** | 0.6151 | GDP → FDI |
| Sweden | 0.4262 | 0.1474 | GDP ⇹ FDI |
| Switzerland | -0.6743 | -0.5657 | GDP ⇹ FDI |
| **Emerging** | **-0.9750** | **1.3790** | **GDP ⇹ FDI** |
| Greece | -0.2937 | -0.2443 | GDP ⇹ FDI |
| Hungary | -0.7071 | 0.2065 | GDP ⇹ FDI |
| Poland | -0.6879 | 2.4263** | FDI → GDP |
| **Frontier** | **-0.8157** | **1.8608*** | **FDI → GDP** |
| Iceland | -0.5912 | -0.0908 | GDP ⇹ FDI |
| Romania | 0.0999 | 0.2705 | GDP ⇹ FDI |
| Serbia | -0.6906 | 0.6337 | GDP ⇹ FDI |
| Slovenia | -0.4496 | 2.9084*** | FDI → GDP |
| **Mediterranean** |  |  |  |
| **Emerging** | **-0.9014** | **1.0437** | **GDP ⇹ FDI** |
| Saudi Arabia | -0.6777 | -0.0591 | GDP ⇹ FDI |
| United Arab Emirates | -0.5970 | 1.5352 | GDP ⇹ FDI |
| **Frontier** | **0.0776** | **-0.4427** | **GDP ⇹ FDI** |
| Oman | 0.0776 | -0.4427 | GDP ⇹ FDI |
| **Oceanian** |  |  |  |
| **Developed** | **-0.6454** | **-0.4537** | **GDP ⇹ FDI** |
| Australia | -0.6806 | -0.3804 | GDP ⇹ FDI |
| New Zealand | -0.2322 | -0.2613 | GDP ⇹ FDI |

Note: *Reject H_0_ at a 10% level of significance; **Reject H_0_ at a 5% level of significance; ***Reject H_0_ at a 1% level of significance. ⇹ denotes nondirectional relationship, → denotes unidirectional relationship, ↔ denotes bidirectional relationship.

Source: Authors’ Calculations
