# Supplementary material for: More than just investment: Causality analysis between foreign direct investment and economic growth
Source: PLoS One. 2022 Nov 3;17(11):e0276621. doi: 10.1371/journal.pone.0276621 (PMC9632898; doi:10.1371/journal.pone.0276621)
Supplement: S4 Appendix — (DOCX) [file pone.0276621.s004.docx]

**S4 Appendix:** **Results of panel regression for global and European region with European sovereign debt crisis as a recession**

**S4.1: GDP as dependent variable**

|  |  | **Global** | **Europe** |
| --- | --- | --- | --- |
| **Variable** |  | **GDP** | **GDP** |
| FDI | | -2.23** | -2.21** |
|  | | (5.09) | (15.35) |
| Recession | | -1.43 | -0.89 |
|  | | (409.18) | (728.98) |
| Constant | | 1.45 | 1.37 |
|  | | (70.77) | (238.90) |
| No. of observations | | 1170 | 270 |
| No. of countries | | 117 | 27 |
| No. of years | | 10 | 10 |
| R^2^ Within | | 0.0064 | 0.0209 |
| R^2^ Between | | 0.0046 | 0.0243 |
| R^2^ Overall | | 0.0059 | 0.0209 |

Note: **Reject at a 5% level of significance. Parentheses represent the robust standard error.

Source: Authors’ Compilation

**S4.2: FDI as dependent variable**

|  | **Global** | **Europe** |
| --- | --- | --- |
| **Variable** | **FDI** | **FDI** |
| GDP | -2.23** | -2.21** |
|  | (<0.00) | (<0.00) |
| Recession | -0.79 | 0.05 |
|  | (2.35) | (2.88) |
| Constant | -0.47 | 0.18 |
|  | (0.41) | (0.95) |
| No. of observations | 1170 | 270 |
| No. of countries | 117 | 27 |
| No. of years | 10 | 10 |
| R^2^ Within | 0.0043 | 0.0193 |
| R^2^ Between | 0.0208 | 0.0019 |
| R^2^ Overall | 0.0047 | 0.0181 |

Note: **Reject at a 5% level of significance. Parentheses represent the robust standard error.

Source: Authors’ Compilation
